# Niraparib Suppresses Cholangiocarcinoma Tumor Growth by Inducing Oxidative and Replication Stress

**DOI:** 10.3390/cancers13174405

**Published:** 2021-08-31

**Authors:** Vladimir Bezrookove, John M. Patino, Mehdi Nosrati, Pierre-Yves Desprez, Sean McAllister, Liliana Soroceanu, Ari Baron, Robert Osorio, Mohammed Kashani-Sabet, Altaf A. Dar

**Affiliations:** California Pacific Medical Center Research Institute, 475 Brannan St., Suite 130, San Francisco, CA 94107, USA; bezroov@cpmcri.org (V.B.); patinojm@cpmcri.org (J.M.P.); nosratm@cpmcri.org (M.N.); desprepy@cpmcri.org (P.-Y.D.); mcallis@cpmcri.org (S.M.); sorocel@cpmcri.org (L.S.); abaron@phoamd.com (A.B.); osorior@sutterhealth.org (R.O.); kashani@cpmcri.org (M.K.-S.)

**Keywords:** cholangiocarcinoma, PAPR inhibitors, patient derived xenograft, oxidative stress, replication fork stalling

## Abstract

**Simple Summary:**

Cholangiocarcinoma (CCA) is a rare and highly aggressive tumor with limited therapeutic options, thus underscoring the need to develop novel therapeutic approaches. We analyzed a publicly available CCA patient database to identify mutations in DNA damage response (DDR) genes. Mutations in DDR genes were prevalent, thus rendering these tumors potentially susceptible to poly-ADP-ribose polymerase (PARP) inhibition. PARP genes are critical to DNA repair and genomic stability. The role of PARP inhibitors in CCA was investigated by employing a series of in vitro functional assays and in vivo patient-derived xenograft models. This study highlights the therapeutic potential of PARP inhibitors alone or in combination with the chemotherapeutic agent gemcitabine for the treatment of CCA.

**Abstract:**

Cholangiocarcinoma (CCA) is the second most common hepatobiliary cancer, an aggressive malignancy with limited therapeutic options. PARP (poly (ADP-ribose) polymerase) 1 and 2 are important for deoxyribonucleotide acid (DNA) repair and maintenance of genomic stability. PARP inhibitors (PARPi) such as niraparib have been approved for different malignancies with genomic alteration in germline *BRCA* and DNA damage response (DDR) pathway genes. Genomic alterations were analyzed in DDR genes in CCA samples employing The Cancer Genome Atlas (TCGA) database. Mutations were observed in various DDR genes, and 35.8% cases had alterations in at least one of three genes (*ARID1A*, *BAP1* and *ATM*), suggesting their susceptibility to PARPi. Niraparib treatment suppressed cancer cell viability and survival, and also caused G2/M cell cycle arrest in patient-derived xenograft cells lines (PDXC) and established CCA cells harboring DDR gene mutations. PARPi treatment also induced apoptosis and caspase3/7 activity in PDXC and CCA cell lines, and substantially reduced expression of BCL2, BCL-XL and MCL1 proteins. Niraparib caused a significant increase in oxidative stress, and induced activation of DNA damage markers, phosphorylation of CHK2 and replication fork stalling. Importantly, niraparib, in combination with gemcitabine, produced sustained and robust inhibition of tumor growth in vivo in a patient-derived xenograft (PDX) model more effectively than either treatment alone. Furthermore, tissue samples from mice treated with niraparib and gemcitabine display significantly lower expression levels of pHH3 and Ki-67, which are a mitotic and proliferative marker, respectively. Taken together, our results indicate niraparib as a novel therapeutic agent alone or in combination with gemcitabine for CCA.

## 1. Introduction

Cholangiocarcinoma (CCA) is a highly aggressive biliary tract cancer (BTC) that accounts for 10–20% [1] of all hepatobiliary malignancies. CCA is the second most common hepatic cancer with poor outcomes, [2] with a 5-year overall survival rate of less than 5% [3]. Both the incidence and mortality of CCA have been increasing rapidly worldwide [4,5,6]. Although curative resection is an effective treatment for CCA, a significant proportion of patients have advanced stage CCA at presentation, and thus are not amenable to resection [2]. Due to the dearth of treatment alternatives and its poor prognosis, there is a pressing need to develop novel therapeutic approaches for the treatment of CCA. 

CCA exhibit alterations in known tumor suppressors and oncogenic drivers, suggesting the possibility of targeted therapies in a subset of patients [7,8,9]. Activating FGFR pathway genomic alterations are present in around 20% of CCAs [7,9,10,11]. Genomic analysis of BTCs indicates that nearly 40% of patients possess potential targetable genetic alterations, focused on DNA damage repair pathway deficiency, cell cycle deregulation and genomic instability [12]. Cancer cells are dependent on compromised DNA damage response (DDR) for their survival due to accumulative DNA damage and chronic replication stress. Thus, targeting DDR and repair pathways has emerged as a promising therapeutic anticancer approach [13,14]. The PARP (poly (ADP-ribose) polymerase) family of enzymes includes PARP1, PARP2 and PARP3 [15]. PARP1, and to a lesser extent PARP2, are essential for DNA repair via the base excision repair pathway and in the maintenance of genomic stability [16]. PARPs detect single-strand DNA breaks (SSBs) and initiate repair [17], as well as being involved in base excision and double-strand DNA break repair [18]. Thus, inhibiting PARP activity leads to unrepaired SSBs and accumulation of stalled replications forks, resulting in the formation of lethal DNA double-strand breaks [19] that are preferentially repaired by homologous recombination (HR)-mediated repair (HRR) pathways [16,18]. Due to the critical role of PARPs, targeting them with specific inhibitors represents an attractive and rational therapeutic strategy. Several PARPi, including niraparib, olaparib, rucaparib and talazoparib are approved by the U.S. FDA. PARPi are in clinical use for the treatment of breast and ovarian cancers [20], and their use has been extended to prostate and pancreatic cancers [21,22]. PARPi monotherapy has shown limited clinical benefit, as most cancers are HR-proficient. Thus, combining them with other targeted or chemotherapeutic agents may result in improved clinical benefit. PARP inhibition has not been extensively studied in CCA patients, and data regarding the role of PARPi in BTC patients possessing BRCA and DDR mutations is sparse [23,24,25].

In this study, we investigated the effects of PARPi (niraparib) therapy on CCA using cell lines and novel PDX models harboring DDR mutations. Niraparib treatment induced apoptosis, oxidative stress, replication fork stalling, and in combination with gemcitabine, produced sustained and robust in vivo antitumor activity. Taken together, this study suggests niraparib alone or in combination with gemcitabine as an effective clinical therapy for CCA patients.

## 2. Results

### 2.1. Genetic Alterations in DDR Genes in CCA

The role of DDR genomic alterations in CCA pathogenesis is largely unexplored, even though efforts are underway to identify a patient population with these mutations that may benefit from personalized targeted therapies. We analyzed the publicly available TCGA database available on the cBioportal site (cbioportal.org, accessed on 25 April 2021) to identify the mutations in a panel of DDR genes (*ARID1A*, *ARID1B*, *ATM*, *ATR*, *ATRX*, *BAP1*, *BARD1*, *BLM*, *BRCA1*, *BRCA2*, *BRIP1*, *CHEK2*, *FANCA/C/D2/E/F/G/L*, *MRE11*, *NBN*, *PALB2*, *RAD50*, *RAD51*, *RAD51B/C* and *WRN)* in CCA samples (*n* = 195). Genomic alterations were observed in *ARID1A* (20.51%), *BAP1* (13.3%) and *ATM* (7.7%), followed by *ARID1B* (2.6%), *BRCA1* (2.1%) and *ATR* (2.1%), as shown in Table S1. Mutations in at least one of the most commonly altered DDR genes (*ARID1A*, *BAP1* and *ATM)* were observed in 35.8% of cases. These findings indicate that a subset of CCA samples harbor alterations in *BRCA* and/or in DDR genes, making them suitable for testing sensitivity to PARP inhibition. 

### 2.2. PARP Inhibitors Suppress Cell Survival and Proliferation

Four CCA cell lines, 2 established (KMCH-1 and HuCCT1) and 2 PDXC (CHNG6 and CHNG31, established in our laboratory from patient samples) each harboring mutations in DDR genes (as mentioned in the Methods section) were employed for measuring response to PARPi. Two widely studied PARPi (niraparib and olaparib) were employed in this study. In addition, olaparib is the only PARPi approved for pancreatic cancer, another gastrointestinal malignancy.

**Figure 1:**
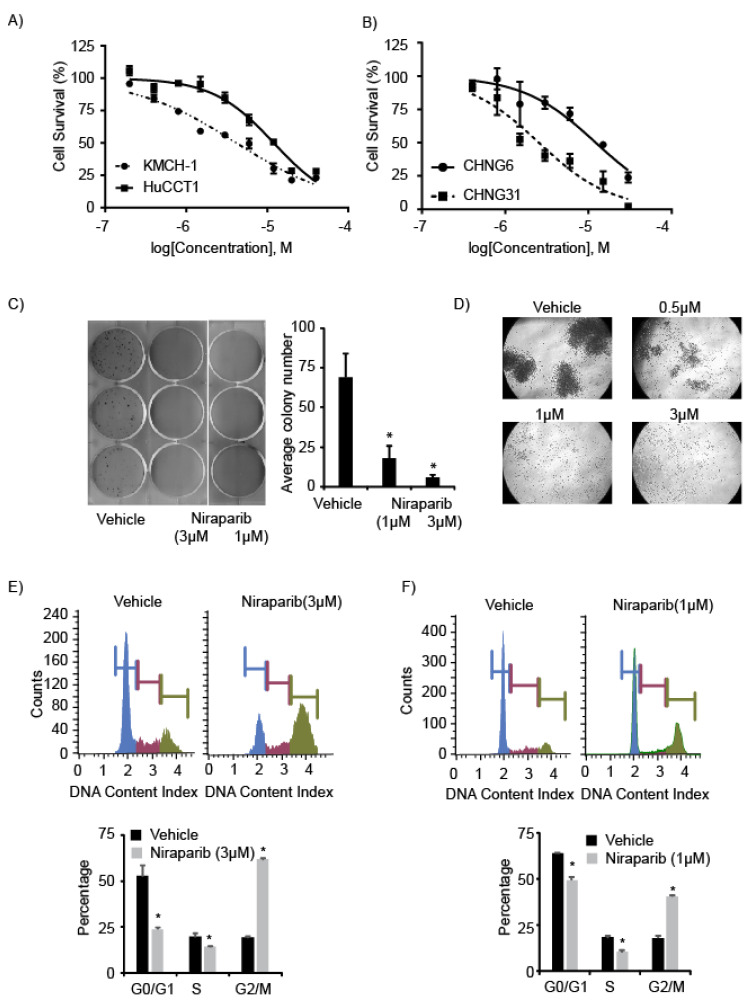
Effects of niraparib treatment on tumor cell survival and proliferation: (**A**) Cell viability analysis of established CCA cell lines (KMCH-1, HuCCT1) after niraparib treatment for 72 h. (**B**) Cell viability of patient derived xenograft lines (CHNG6, CHNG31) after niraparib treatment for 72 h. (**C**) Colony formation ability of KMCH-1 cells after niraparib treatment. Bar graph showing average colony number after different treatments. (**D**) Spheroid formation of CHNG31 after treatment with various concentrations of niraparib. Cell cycle analysis of (**E**) KMCH-1 or (**F**) CHNG31 after niraparib treatment for 72 h. Bar graphs showing analysis of different cell cycle phases after vehicle or niraparib treatment. * *p* < 0.05. *p* values were calculated using the two-sided Student’s *t* test and data presented as mean + stdev.

Niraparib substantially suppressed the cell survival of established CCA cell lines and PDXC, with an IC50 range of 1–10 µM (Figure 1A,B), while CCA cells were less sensitive to olaparib (Figure S1A). Niraparib treatment reduced the colony formation ability of KMCH-1 cells, as evidenced by the reduced colony number and size (Figure 1C and Figure S1B). Spheroid formation of CHNG31 cells was substantially reduced with niraparib treatment (Figure 1D and Figure S1C). Niraparib treatment significantly induced G2/M arrest and also resulted in a reduced S-phase population when compared to vehicle (DMSO) treatment in KMCH-1 and CHNG31 cells (Figure 1E,F and Figure S1D,E). Treatment of KMCH-1 cells with olaparib also resulted in G2/M phase arrest, albeit requiring a higher dose (Figure S1F). These observations indicate that PARPi can significantly suppress CCA cell proliferation.

### 2.3. Niraparib Induces Apoptosis and Suppress Expression of Anti-Apoptotic Proteins

**Figure 2:**
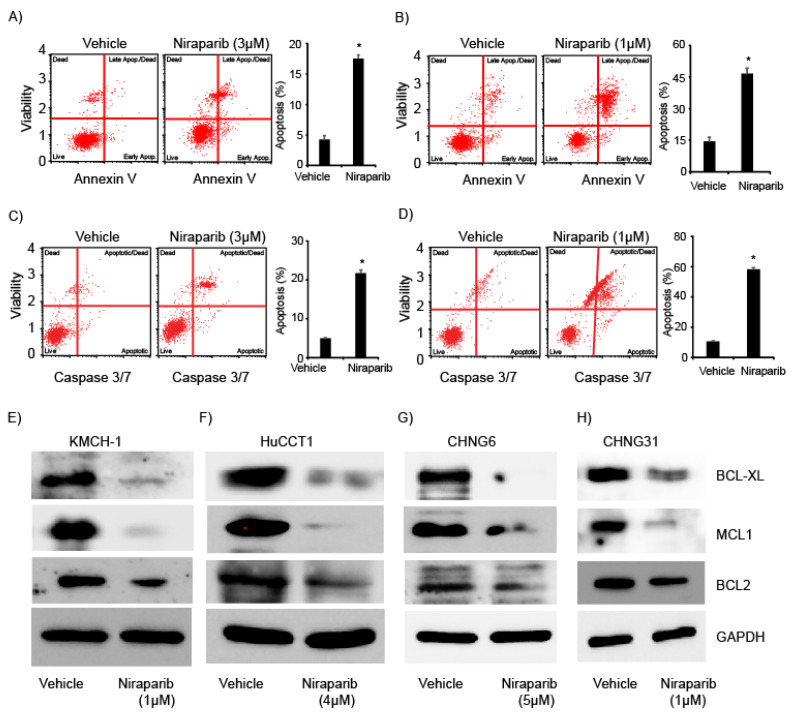
Effects of niraparib treatment on apoptosis, caspase3/7 activity and anti-apoptotic proteins: Apoptosis index of (**A**) KMCH-1 or (**B**) CHNG31 after niraparib treatment for 72 h. Bar graphs showing the percentage of apoptotic cells after vehicle or niraparib treatment. Caspase3/7 activity analysis of (**C**) KMCH-1 or (**D**) CHNG31 after niraparib treatment for 72 h. Bar graphs showing the percentage of caspase3/7 activity after vehicle or niraparib treatment. (**E**–**H**) Western blot analysis of BCL2, BCL-XL and MCL1 after niraparib treatment for 48 h in KMCH-1, HuCCT1, CHNG6 and CHNG31, respectively. * *p* < 0.05. *p* values were calculated using the two-sided Student’s *t* test and data presented as mean + stdev.

Next, we studied the effects of niraparib on apoptosis of established CCA and PDXC lines. Niraparib treatment induced an increase in the annexin V-positive apoptotic cell population in KMCH-1 and CHNG31 cell lines (Figure 2A,B and Figure S2A,B). We also observed enhanced caspase3/7 activity following niraparib treatment when compared to vehicle treatment in KMCH-1 and CHNG31 cell lines (Figure 2C,D and Figure S2C,D). These observations indicate the ability of niraparib to increase the apoptotic index in established and patient-derived xenograft CCA cells in culture. By contrast, treatment of KMCH-1 cells with olaparib had a minimal effect on apoptosis induction (Figure S2E). We also determined the effects of niraparib treatment on the expression of anti-apoptotic BCL2 protein family members. Niraparib treatment suppressed the expression of BCL2, BCL-XL and MCL1 in two established and two PDXC lines (Figure 2E–H). Suppression of anti-apoptotic proteins and increased caspase 3/7 activity may explain the observed reduction in cell growth and apoptosis observed in CCA cells. 

### 2.4. Niraparib Induces DNA Damage, Oxidative Stress, and Replication Fork Stalling

**Figure 3:**
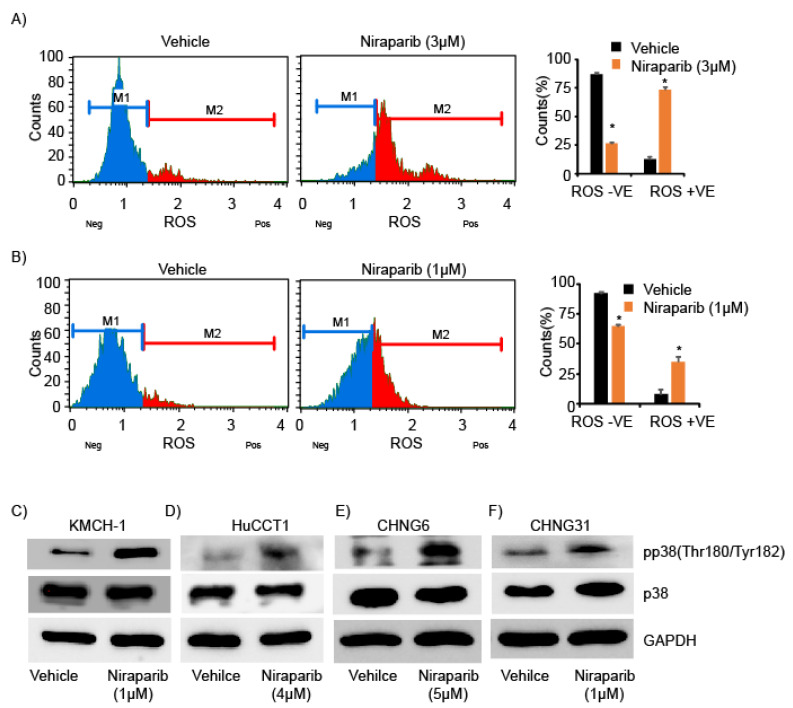
Effects of niraparib on oxidative stress and phosphorylation of p38MAPK: Analysis of oxidative stress in (**A**) KMCH-1 or (**B**) CHNG31 after niraparib treatment for 72 h. Bar graphs showing percentage of ROS-VE and ROS+VE populations after drug treatment. (**C**–**F**) Western blot analysis of phosphorylation of p38MAPK after niraparib treatment for 48 h in KMCH-1, HuCCT1, CHNG6 and CHNG31 cells, respectively. * *p* < 0.05. *p* values were calculated using the two-sided Student’s *t* test and data presented as mean + stdev.

**Figure 4:**
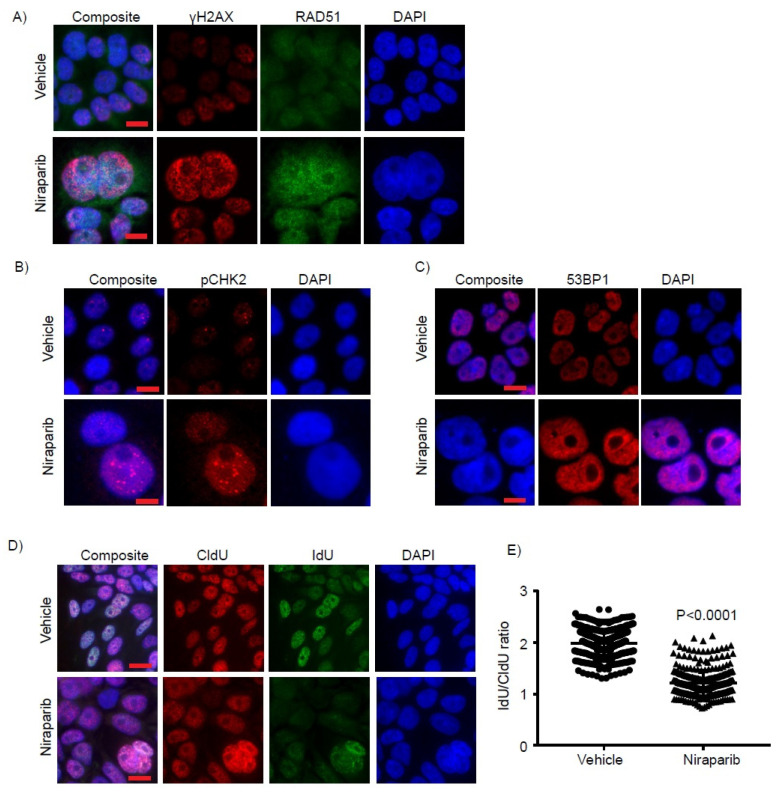
Niraparib induces DNA damage and replication fork stalling: Representative immunofluorescence images of (**A**) γH2AX, RAD51, (**B**) pCHK2 and (**C**) 53BP1 of KMCH-1 cells treated with niraparib for 72 h. (**D**) Representative images of CldU and IdU positive KMCH-1 cells treated with niraparib for 48 h. (**E**) Scatter plot showing ratio of IdU/CldU intensities in KMCH-1 cells treated with niraparib. Scale bar = 20 µm. The *p* value for panel E was calculated using the two-sided Student’s *t* test and data presented as mean + stdev.

We next assessed whether niraparib treatment results in oxidative DNA damage and stress. Treatment of KMCH-1 and CHNG31 cell lines with niraparib substantially increased reactive oxygen species (ROS) levels in drug-treated cells (Figure 3A,B and Figure S3A,B). KMCH-1 cells treated with olaparib also showed enhanced ROS levels (Figure S3C). We observed increased phosphorylation of p38MAPK after niraparib treatment (Figure 3C–F), which is known to occur in response to oxidative stress [26]. Niraparib treatment induced increased expression and activation of the DNA damage marker γH2AX in KMCH-1 and HuCCT1 cell lines (Figure 4A, Figures S4A and S5A,B). Consistent with increased γH2AX levels, expression of RAD51, which is recruited by γH2AX, was also upregulated (Figure 4A, Figures S4A and S5C,D). Phosphorylated CHK2 protein levels were significantly increased after niraparib treatment (Figure 4B, Figures S4B and S5E,F), indicating activation of ATM-CHK2 DDR pathways. Expression of 53BP1, another member of the DDR response, was substantially increased with niraparib treatment (Figure 4C, Figures S4C and S5G,H). We further analyzed the effects of niraparib treatment on DNA replication and fork progression, as the aforementioned proteins are key mediators of replication initiation and fork stability. KMCH-1 and HuCCT1 cell lines were exposed to 5-chloro-2′-deoxyuridine (CldU) for 20 min, followed by 48 h niraparib treatment, and then pulsed for 20 min with Idoxuridine (IdU). While the CldU incorporation rate was similar between the vehicle and niraparib treatment groups, a significant drop in the population of IdU-positive cells was observed in the niraparib group, reflecting severe replication fork destabilization (Figure 4D,E and Figure S4D,E). These observations suggest that PARP inhibition causes oxidative stress, DNA damage and replication fork stalling in CCA cells.

### 2.5. Niraparib in Combination with Gemcitabine Suppressed In Vivo Tumor Growth

**Figure 5:**
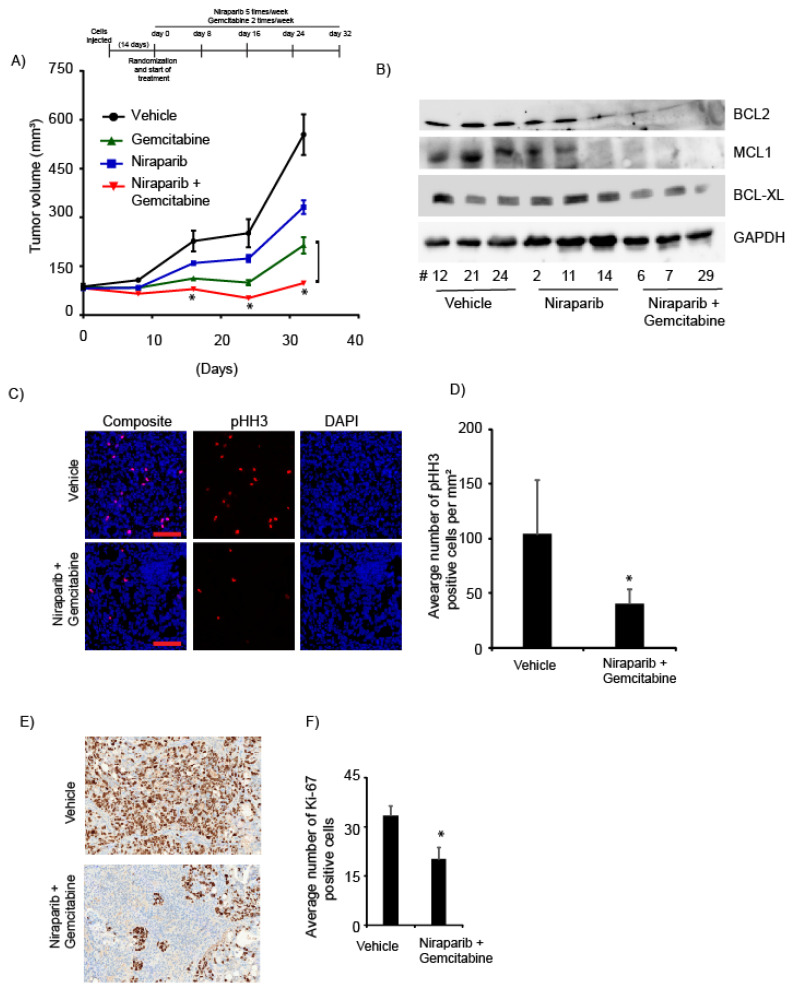
Effect of niraparib on in vivo tumor growth: (**A**) Niraparib in combination with gemcitabine significantly enhanced suppression of in vivo tumor growth compared with either of the treatments alone [for vehicle (*n* = 7), niraparib (*n* = 7), gemcitabine (*n* = 7) and niraparib plus the gemcitabine combination group (*n* = 10)]. Palpable tumors were observed after 14 days of cell implantation. Graphics indicate dosing schedule. (**B**) Western blot analysis showing expression of BCL2, BCL-XL and MCL1 from in vivo tumor samples. (**C**) Representative immunofluorescence (IF) images showing pHH3 positively stained cells (red) from the vehicle and combination-treated groups. Bar graph showing average number of positive pHH3 cells per mm^2^ in three samples each from the vehicle and combination-treated groups. (**D**) Representative immunohistochemical (IHC) images showing Ki-67 staining in the vehicle and combination-treated samples. Bar graph representing the average number of Ki-67 positive cells in three samples each from the vehicle and combination-treated samples. Analysis of Ki-67, a pro-proliferative marker, was suppressed in the combination-treated group when compared to vehicle control (**E**,**F**). Scale bar; IF = 100 µm and IHC = 100 µm. * *p* < 0.05. *p* values for panel D and F were calculated using the two-sided Student’s *t* test and data presented as mean + stdev.

Next, we investigated the effects of niraparib alone and in combination with gemcitabine on in vivo CCA tumor growth in the CHNG31 PDX. Niraparib and gemcitabine treatment alone reduced tumor growth (Figure 5A). However, niraparib, in combination with gemcitabine, suppressed tumor growth to a greater extent when compared to either of the single agents (Figure 5A). Analysis of proteins extracted from tumors treated with the combination exhibited reduction in expression levels of anti-apoptotic proteins (BCL2, BCL-XL and MCL1) when compared to vehicle- or niraparib-treated samples (Figure 5B). Analysis of in vivo drug-treated tumors revealed significant suppression of the mitotic marker phosphorylated histone H3 (Ser10) (pHH3), in the combination treatment group (Figure 5C,D). Finally, analysis of Ki-67, a pro-proliferative marker, was suppressed in the combination-treated group when compared to vehicle control (Figure 5E,F). These observations indicate the effectiveness of the combination treatment in suppressing CCA in vivo tumor growth.

## 3. Discussion

CCA is a rare malignancy with a dismal prognosis, which is responsible for 10–20% of hepatic malignancy-related deaths [27]. CCA patients typically present at an advanced stage, with radical surgery being the only curative treatment option. Systemic chemotherapy with cisplatin in combination with gemcitabine represents the standard of care, but the majority of patients develop progressive disease. No targeted therapy has been approved to treat CCA, highlighting an urgent need to identify new therapeutic modalities. Here, we report the pre-clinical significance of PARPi (niraparib) alone or in combination with gemcitabine in CCA harboring mutations in DDR genes, employing both established cell lines and novel PDX lines. 

The genomic characterization of different tumor types has significantly increased the potential to identify targetable oncogenic alterations. Analysis of a publicly available database for potential alterations in *BRCA* and additional DDR genes in CCA patient samples identified mutations in several genes, indicating their potential sensitivity to PARPi. Genomic alterations were predominantly detected in *ARID1A* (20.51%), *BAP1* (13.3%) and *ATM* (7.7%). Additionally, 35.8% of CCA samples harbored a mutation in at least one of three DDR genes (*ARID1A*, *BAP1* or *ATM).* Mutations in *ARID1A* have been previously reported in up to 14% of CCA samples [28] and its deficiency hypothesized to sensitize cancer cells to PARPi [29]. *BAP1*, a tumor suppressor and a deubiquitinase promoting DNA DSB repair, is also involved in HR [30] and CCAs harboring *BAP1* mutations are likely to have a poorer prognosis [31]. *BRCA1/2* mutations were observed in 1–2% CCA samples, and Rizzo et al. [12] reported 1–7% of *BRAC1/2* mutations in BTC samples, associated with poor response to standard treatments. To date, clinical data regarding PARP inhibition in BTC and in particular in CCA harboring *BRCA* and DDR mutations are sparse, and few sporadic cases of response to PARP inhibition have been reported [24,25]. The PARP enzymes sense DNA strand breaks and play key roles in base excision repair for repairing single strand breaks (SSB) and also recruit DNA repair proteins [32,33,34]. Double strand breaks (DSB) are generated if SSB are not repaired. Any defect in the repair process promotes mutagenesis and tumorigenesis. As a result, targeting such vulnerabilities has emerged as a selective and rational anticancer strategy. Several PARPi have been approved for the treatment of *BRCA1/2*- mutated ovarian and breast cancers, and have now been also approved for prostate and pancreatic cancers. A number of other PARPi are in different stages of preclinical and clinical development [20,35]. While PARPi are reported to have comparable inhibition of PAPR1 and PARP2 [36,37], they differ in the capacity to induce DNA strand break, apoptosis and PARP trapping [38,39]. Niraparib and olaparib, FDA-approved PARPi, were employed for the treatment of CCA cells. Niraparib treatment resulted in significant suppression in cell survival and proliferation in CCA cell lines and PDXC lines. Niraparib and olaparib treatment promoted G2/M arrest, in addition to decrease in the S-phase of the cell cycle. The colony and spheroid formation abilities of CCA and PDXC cells were drastically curtailed by niraparib treatment. These results are in agreement with previous reports of PARPi suppressing cellular proliferation of ovarian cancer [40]. Increased caspase3/7 activity, along with induction of apoptosis, was observed with niraparib treatment, and accompanied by suppressed expression of BCL2, BCL-XL and MCL1. We have previously reported overexpression of BCL-XL in CCA samples [41], suggesting that samples with BCL-XL overexpression may be sensitive to PARPi.

Reactive oxygen species (ROS) in cancer cells induce mutations in oncogenic pathways that drive cancer progression [42]. However, higher levels of ROS may lead to cell death, with an important role in suppressing cancer initiation and progression [43,44]. Cancer cells are dependent on antioxidant systems and DNA repair for survival due to abnormal metabolism and oxidative pressure, thus rendering them sensitive to oxidative insults by enhancing ROS levels [45]. Niraparib and olaparib treatment induced a rapid increase in ROS levels, with niraparib inducing substantially higher ROS levels than olaparib. ROS induction was accompanied by increase in the phosphorylation of p38MAPK, which is known to occur due to oxidative stress [26]. These observations indicate that PARPi can elevate oxidative stress and induce oxidative DNA damage, rendering CCA cells susceptible to drug treatment. Niraparib treatment markedly increased levels of γH2AX, RAD51, 53BP1, and pCHK2. In addition, CldU and IdU labeling revealed severe replication fork stalling following niraparib treatment. Increases in 53BP1 and γH2AX indicate the presence of extensive DSBs, DNA damage checkpoint defects and impaired DNA repair [46]. Increases in the phosphorylation of CHK1 or CHK2 transduce DNA damage signals through a phosphorylation cascade involving ATR and ATM, respectively. Activation of the CHK2-ATM axis mediates G2/M cell cycle arrest [47,48] suggesting niraparib-mediated CHK2 activation as a possible mechanism for the G2/M cell cycle arrest observed in CCA.

Niraparib, in combination with gemcitabine, significantly suppressed the in vivo tumor cell growth of a CCA PDX line, and the combination therapy was more effective than either of the agents alone. In addition, the in vivo study showed substantial suppression of expression of the mitotic marker pHH3 and the proliferative marker Ki-67 in the combination treatment group, confirming the potent anti-proliferative effects of this drug combination. PARP inhibition is currently being investigated for possible synergy with various targeted or immunotherapies in different tumors [49,50,51]. Very little information is available regarding the efficacy of PARPi in BTC patients harboring DDR gene mutations, except for a report demonstrating the clinical benefit of olaparib treatment in a gallbladder cancer patient harboring an *ATM* inactivating mutation [23,52,53]. To date, clinical trials of PARPi in gastrointestinal malignancies have led to the approval of olaparib for BRCA-mutant pancreatic cancer patients, prompting multiple clinical trials evaluating the potential role of PARPi in BTC. Our study is timely in this regard, and strongly suggests the clinical benefit of PARPi in combination with gemcitabine in tumors harboring mutations in one or more DDR genes.

## 4. Material and Methods

### 4.1. Cell Culture and Patient-Derived Xenograft Mouse Models

Patient recruitment and sample acquisition was performed under an Institutional Review Board (IRB) protocol approved at California Pacific Medical Center in accordance with relevant guidelines and regulations. Informed consent was obtained from the patients in accordance with approved institutional guidelines. Patient derived xenograft (PDX) and PDX derived cell (PDXC) generation, cell culture conditions and short tandem repeat (STR) analysis were previously described by our group [41]. Two PDXC designated as CHNG6 (with mutations in *ARID2*, *ATM*) and CHNG31 (with mutations in *BRCA1*, *PALB2* and *ARID1A*) were employed and cultured as spheroids without fetal bovine serum (FBS). The human CCA cell lines KMCH-1 (with a mutation in *ATM*) (kindly provided by Dr. Gregory Gores, Mayo Clinic, MN, USA) and HuCCT1 (with mutations in *BRCA2*, *POLE2*) purchased from the Japanese Collection of Research Bioresources Cell Bank (JCRB, Osaka, Japan) were employed. KMCH-1 and HuCCT1 were grown in RPMI (ThermoFisher Scientific, South San Francisco, CA, USA) with 5% fetal bovine serum (JR Scientific, Woodland, CA, USA) and 1× penicillin/streptomycin (Thermofisher Scientific) at 37 °C in a 5% CO_2_ incubator. Cell lines tested negative for mycoplasma contamination using MycoFluor Mycoplasma Detection Kit (Thermofisher Scientific) following the manufacturer’s instructions.

### 4.2. TCGA Dataset for DDR Mutation Analysis

Mutations in the following DDR genes: *ARID1A*, *ARID1B*, *ATM*, *ATR*, *ATRX*, *BAP1*, *BARD1*, *BLM*, *BRCA1*, *BRCA2*, *BRIP1*, *CHEK2*, *FANCA/C/D2/E/F/G/L*, *MRE11*, *NBN*, *PALB2*, *RAD50*, *RAD51*, *RAD51B/C* and *WRN* were analyzed in CCA samples (*n* = 195) employing cBioportal database (cbioportal.org, accessed on 25 April 2021).

### 4.3. Colony Formation, Spheroid Assay and Drugs

To determine colony formation ability, 300–500 established CCA cells were plated in each well of a 6-well plate as described previously [41]. Drugs were added 24 h later and cells were allowed to grow until colonies appeared. Crystal violet (Sigma-Aldrich, St. Louis, MO, USA) was used to stain the colonies. The number of colonies was counted and the data were presented as a bar graph. For spheroid formation in PDXC, 1000 cells per well were plated, treated with the indicated drugs and allowed to grow for 6 days. Niraparib, olaparib and gemcitabine were purchased from Selleck chemicals (Houston, TX, USA). 

### 4.4. Cell Survival Assay 

Cell survival was assessed as described [41]. CCA cells (1000–2000) were plated in a 96-well plate and treated the following day with either niraparib or olaparib for 72 h. Cell survival was evaluated by employing the Cell Counting Kit-8 (Dojindo Molecular Technologies, Rockville, MD, USA) following the manufacturer’s instructions. Absorbance was read at 450 nm.

### 4.5. Immunofluorescence and Immunohistochemistry

Immunofluorescence (IF) analysis was performed as described earlier [54]. Specific antibodies for γH2AX (#05-636; 1:100 Millipore Sigma), 53BP1 (#4937; 1:500), pChk2 (#2197; 1:500) and RAD51 (#8875S; 1:500, Cell Signaling) were used. The phosphohistone H3 (Ser10) (#9701; 1:500) IF for in vivo tissue samples was performed as described previously [55]. DAPI staining was used as counterstain. Images were taken at fixed exposures with a Zeiss Axio Image Z2 microscope and the fluorescence intensities of at least 200 cells quantified using Axiovision software version 4.8.2 SP2.

Immunhistochemical (IHC) analysis was performed as described earlier [55,56]. Briefly, Ki-67 IHC was performed by employing Ventana Benchmark autostainer (Ventana Medical Systems, Tucson, AZ, USA) using CONFIRM anti-Ki-67 (30-9) antibody (Ventana Medical Systems).

### 4.6. Oxidative Stress, Annexin V, Cell Cycle and Caspase 3/7 Assays

Oxidative stress, Annexin V, Cell cycle and Caspase 3/7 assays were performed by using the Muse Oxidative Stress kit, Muse Cell Cycle kit, Muse Annexin V Apoptosanis kit and Muse Caspase 3/7 kit, respectively (EMD Millipore, Hayward, CA, USA) following the manufacturer’s instructions and as described previously [55]. 

### 4.7. Pulse-Labeling of DNA Replication by CldU and IdU

CCA cells were labeled with 250 µM 5-chloro-2′-deoxyuridine (CldU) for 20 min, media was replaced and cells incubated with or without niraparib for 48 h, followed by incubation with fresh media containing 25 µM Idoxuridine (IdU) for 20 min. Cells were fixed in 4% formaldehyde for 15 min, followed by treatment with 2M HCl for 20 min. Cells were treated with 0.02% Triton-X 100 in PBS for 10 min followed by blocking with 3% BSA for 10 min and incubated with primary (anti-CldU and anti-IdU) and secondary antibodies. Images were taken at fixed exposures with a Zeiss Axio Image Z2 microscope and the data presented as a ratio of IdU/CldU intensities.

### 4.8. Western Blot Analysis

Protein was extracted from treated cells or tissue samples using RIPA buffer containing 1× Halt phosphatase inhibitor cocktail and 1× Halt protease inhibitor cocktail (Pierce, Rockford, IL). Proteins (10–30 µg) were subjected to SDS/polyacrylamide gel electrophoresis (PAGE) and transferred onto a nitrocellulose membrane. Specific antibodies against BCL2 (#4223; 1:1000), BCL-XL (#2764; 1:1000), p38 MAPK (#9212; 1:1000), pp38 (D3F9 #4511; 1:1000), MCL1 (#39224; 1:1000), and GAPDH (#MAB374; 1:2000) were used. Original blots can be found at Figure S6.

### 4.9. In Vivo Animal Study 

Six- to eight-week-old NSG mice (Strain NOD.Cg-Prkdc^scid^ IL2rg^tm1Wjl^/SzJ) were obtained from Jackson Laboratories (Sacramento, CA). Animal studies were carried out in accordance with the National Institutes of Health guidelines and an approved Institutional Animal Care and Use Committee (IACUC) protocol. PDXC (0.5 × 10^6^) were mixed with 50% Matrigel (without growth factors) in a total volume of 100 µL for subcutaneous injections in the flank. Once tumors were palpable (day 14), mice were randomized and divided into groups with average tumor volumes of 80 mm^3^. Mice were divided into different treatment groups, including a vehicle (*n* = 7), niraparib (*n* = 7), gemcitabine (*n* = 7) and niraparib plus gemcitabine combination group (*n* = 10). Drugs were administered intraperitoneally (i.p.), diluted in 4% DMSO, 4% Tween-80 and 92% saline and the start of the treatment was considered as day 0. Niraparib (25 mg/kg) was administered five days per week and gemcitabine (10 mg/kg) twice weekly each in a volume of 200 µL for four weeks. Toxicity studies were performed to determine the optimal tolerable dose for single and combination drugs. As NSG mice were used in this study which are considered to be more sensitive to drug toxicity, 2–4 mice were used to perform initial toxicity studies to determine whether the chosen doses alone or in combination would be well tolerated before the initiation of a full treatment study. Tumor volumes were measured by caliper and calculated as a product of [(length × width × width)/2]. Tumors were collected and processed for IF, IHC analysis and protein extraction at the end of the study.

### 4.10. Statistical Analysis 

Statistical analyses were performed using GraphPad Prism 7 software (San Diego, CA, USA). Differences in tumor growth between treatment groups were evaluated using two-way ANOVA repeated measures, and a Tukey’s multiple comparisons test. Statistical significance was defined as a *p*-value < 0.05. 

## 5. Conclusions

This study highlights the prevalence of genomic alterations in DDR genes in CCA, as well as the susceptibility of CCA cells harboring such mutations to PARPi. Furthermore, our data demonstrate the utility of niraparib in combination with gemcitabine as a promising therapeutic option for CCA patients.

## Data Availability

The datasets used and/or analyzed during the current study are available from the corresponding author on reasonable request.

## Supplementary Materials

The following are available online at https://www.mdpi.com/article/10.3390/cancers13174405/s1, Figure S1: Effects of olaparib and niraparib treatment on CCA cell survival and proliferation, Figure S2: Effects of niraparib treatment on apoptosis and caspase 3/7 activity, Figure S3: Effects of niraparib treatment on oxidative stress, Figure S4: Niraparib induces DNA damage and replication fork stalling, Figure S5: Immunofluorescence quantification after niraparib treatment, Figure S6: Original Western blots. Table S1: Genomic alterations in Cholangiocarcinoma samples (n = 195) from cBioportal.
