# Supplementary material for: Niraparib Suppresses Cholangiocarcinoma Tumor Growth by Inducing Oxidative and Replication Stress
Source: Cancers (Basel). 2021 Aug 31;13(17):4405. doi: 10.3390/cancers13174405 (PMC8430987; doi:10.3390/cancers13174405)

Supplemental Figure Legends

Figure S1: Effects of olaparib and niraparib treatment on CCA cell survival and proliferation

A) Cell viability analysis of CCA cell lines (KMCH-1, HuCCT1) after olaparib treatment for 72hr. B) Colony formation ability of HuCCT1 cells after niraparib treatment. Bar graph showing average colony number after different treatments. C) Spheroid formation of CHNG6 after treatment with various concentrations of niraparib. Cell cycle analysis of D) HuCCT1or E) CHNG6 after niraparib treatment for 72hr. Bar graphs showing analysis of different cell cycle phases after vehicle or niraparib treatment. F) Cell cycle analysis of KMCH-1 cells treated with olaparib. *P<0.05. P values were calculated using the two-sided Student's t test and data presented as mean+stdev.

Figure S2: Effects of niraparib treatment on apoptosis and caspase 3/7 activity

Apoptosis index of A) HuCCT1 or B) CHNG6 after niraparib treatment for 72hr. Bar graphs showing percentage of apoptotic cells after vehicle or niraparib treatment. Analysis of caspase3/7 activity in C) HuCCT1 or D) CHNG6 cells after niraparib treatment for 72hr. Bar graphs showing percentage of caspase3/7 positive cells after vehicle or niraparib treatment. E) Apoptotic index analysis of KMCH-1 cells after olaparib treatment. *P<0.05. P values were calculated using the two-sided Student's t test and data presented as mean+stdev.

Figure S3: Effects of niraparib treatment on oxidative stress

Analysis of oxidative stress in A) HuCCT1 or B) CHNG6 cells after niraparib treatment for 72hr. Bar graphs showing percentage of ROS-VE and ROS+VE populations after drug treatment. C)

Oxidative stress analysis of KMCH-1 cells treated with olaparib. *P<0.05. P values were calculated using the two-sided Student's t test and data presented as mean+stdev.

Figure S4: Niraparib induces DNA damage and replication fork stalling

Representative immunofluorescence images of A) γH2AX, RAD51, B) pCHK2 and C) 53BP1 of HuCCT1 cells treated with niraparib for 72hr. D) Representative images of CldU and IdU positive HuCCT1 cells treated with niraparib for 48hr. E) Scatter plot showing ratio of IdU/CldU intensities in HuCCT1 cells treated with niraparib. Scale bar=20μm. *P<0.05. P value for panel E was calculated using the two-sided Student's t test and data presented as mean+stdev.

Figure S5: Immunofluorescence quantification after niraparib treatment

Quantitative immunofluorescence quantification of several proteins in KMCH (A, C, E, G) and HuCCT1 cells (B, D, F, H) following niraparib treatment as indicated.

Figure S6: Original Western blots

Table S1

Genomic alterations in Cholangiocarcinoma samples (n=195) from cBioportal

Genes	Cases with mutations (%)
ARID1A	20.51
BAP1	13.3
ATM	7.7
ARID1B	2.6
ATR	2.1
BRCA1	2.1
BRCA2	1
FANCC	1
NBN	1
PALB2	1
ATRX	0.5
BARD1	0.5
BRIP1	0.5
RAD50	0.5
CHEK1	0.5
BLM	0
CHEK2	0
FANCA	0
FANCD2	0
FANCE	0
FANCF	0
FANCG	0
FANCL	0
MRE11	0
RAD51	0
RAD51B	0
RAD51C	0
WRN	0

Figure S1

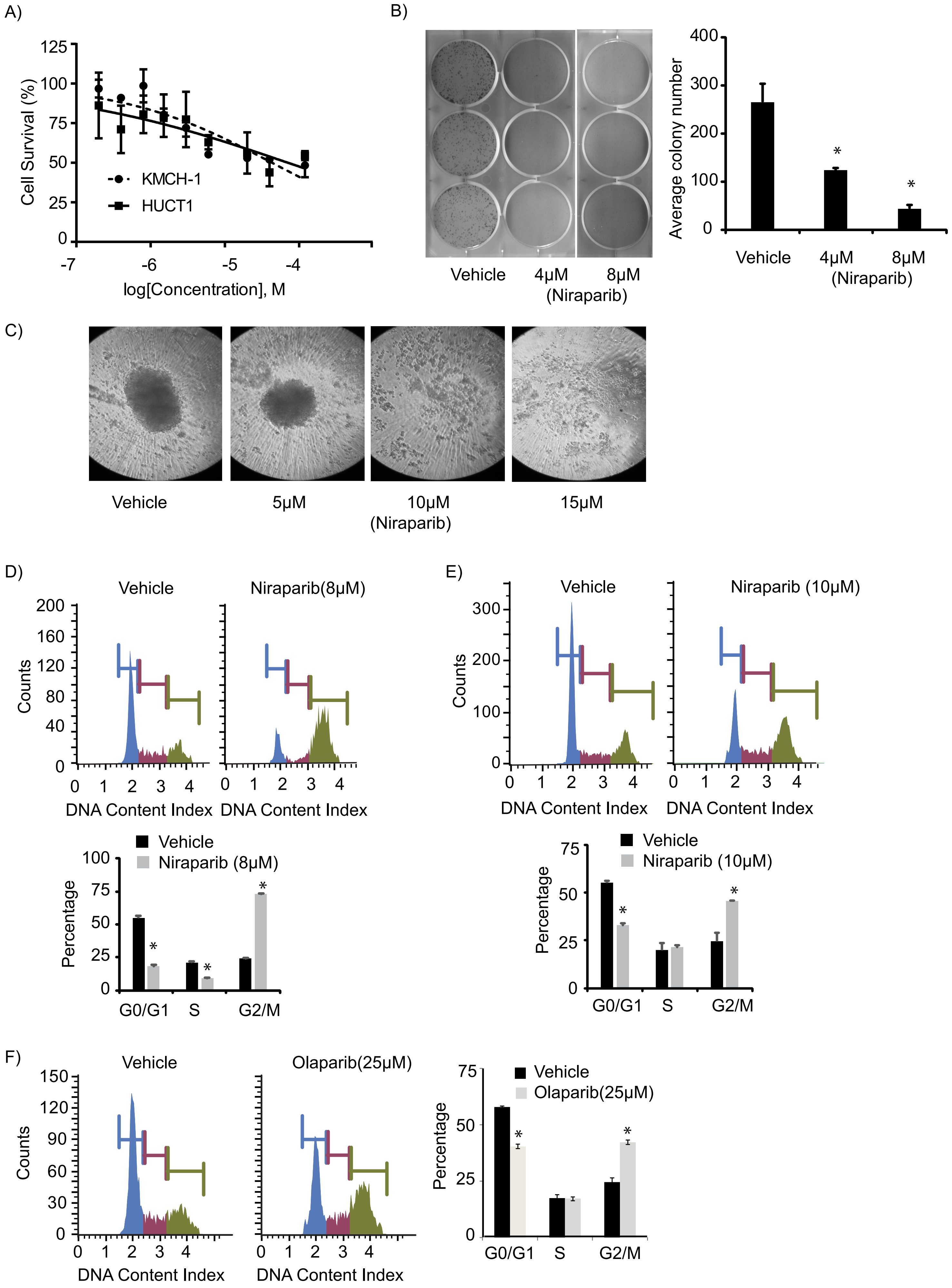
A)
Cell Survival (%)
125
100
75
50
25
0
KMCH-1
HUCT1
-7
-6
-5
-4
log[Concentration], M
B)
Vehicle
4μM
8μM
(Niraparib)
Average colony number
400
300
200
100
0
*
*
Vehicle
4μM
8μM
(Niraparib)
C)
Vehicle
5μM
10μM
(Niraparib)
15μM
D)
Vehicle
Niraparib(8μM)
Counts
200
160
120
80
40
0
0 1 2 3 4
DNA Content Index
DNA Content Index
Vehicle
Niraparib (8μM)
Percentage
100
75
50
25
0
G0/G1
S
G2/M
E)
Vehicle
Niraparib (10μM)
Counts
300
200
100
0
DNA Content Index
DNA Content Index
Vehicle
Niraparib (10μM)
Percentage
75
50
25
0
G0/G1
S
G2/M
F)
Vehicle
Olaparib(25μM)
Counts
150
120
90
60
30
0
DNA Content Index
DNA Content Index
Vehicle
Olaparib(25μM)
Percentage
75
50
25
0
G0/G1
S
G2/M

Figure S2

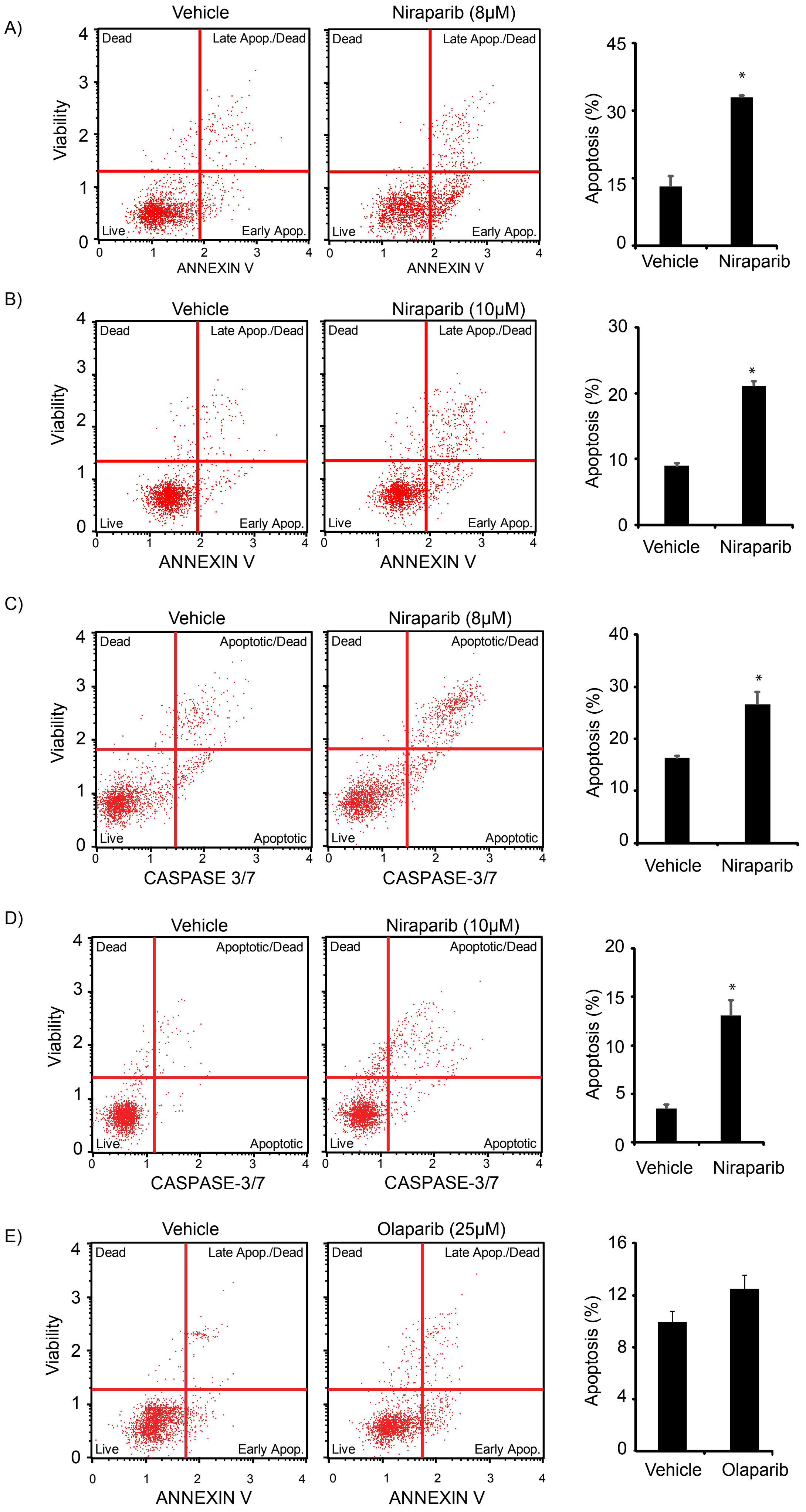
A)
Vehicle
Niraparib (8μM)
Dead
Late Apop./Dead
Live
Early Apop.
Viability
ANNEXIN V
Apoptosis (%)
Vehicle
Niraparib
*
B)
Vehicle
Niraparib (10μM)
Dead
Late Apop./Dead
Live
Early Apop.
Viability
ANNEXIN V
Apoptosis (%)
Vehicle
Niraparib
*
C)
Vehicle
Niraparib (8μM)
Dead
Apoptotic/Dead
Live
Apoptotic
Viability
CASPASE 3/7
CASPASE-3/7
Apoptosis (%)
Vehicle
Niraparib
*
D)
Vehicle
Niraparib (10μM)
Dead
Apoptotic/Dead
Live
Apoptotic
Viability
CASPASE-3/7
Apoptosis (%)
Vehicle
Niraparib
*
E)
Vehicle
Olaparib (25μM)
Dead
Late Apop./Dead
Live
Early Apop.
Viability
ANNEXIN V
Apoptosis (%)
Vehicle
Olaparib

Figure S3

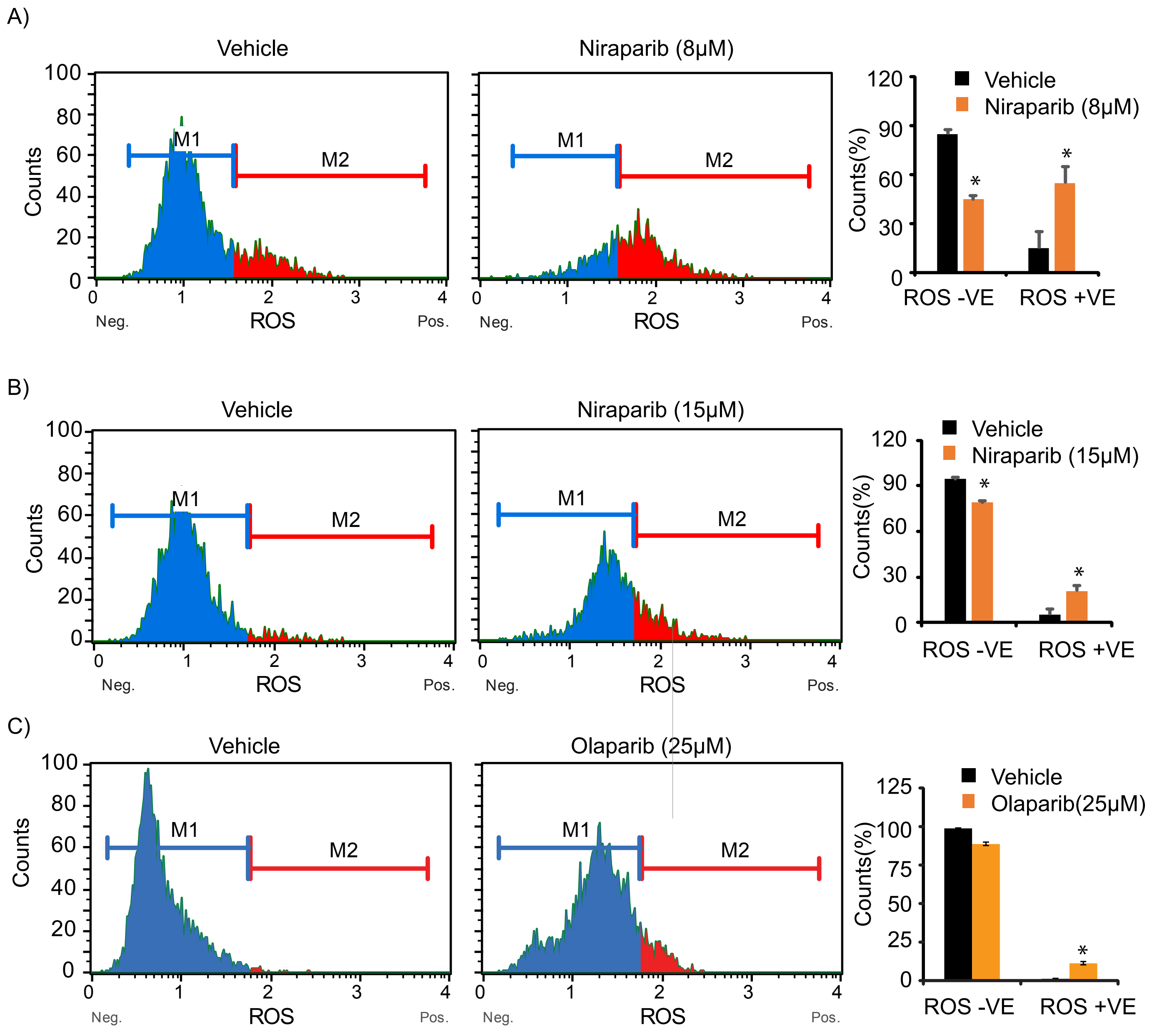

A)
Vehicle
Niraparib (8μM)
Counts
M1
M2
Neg.
ROS
Pos.
Vehicle
Niraparib (8μM)
Counts(%)
ROS -VE
ROS +VE
B)
Vehicle
Niraparib (15μM)
Vehicle
Niraparib (15μM)
C)
Vehicle
Olaparib (25μM)
Vehicle
Olaparib(25μM)

Figure S4

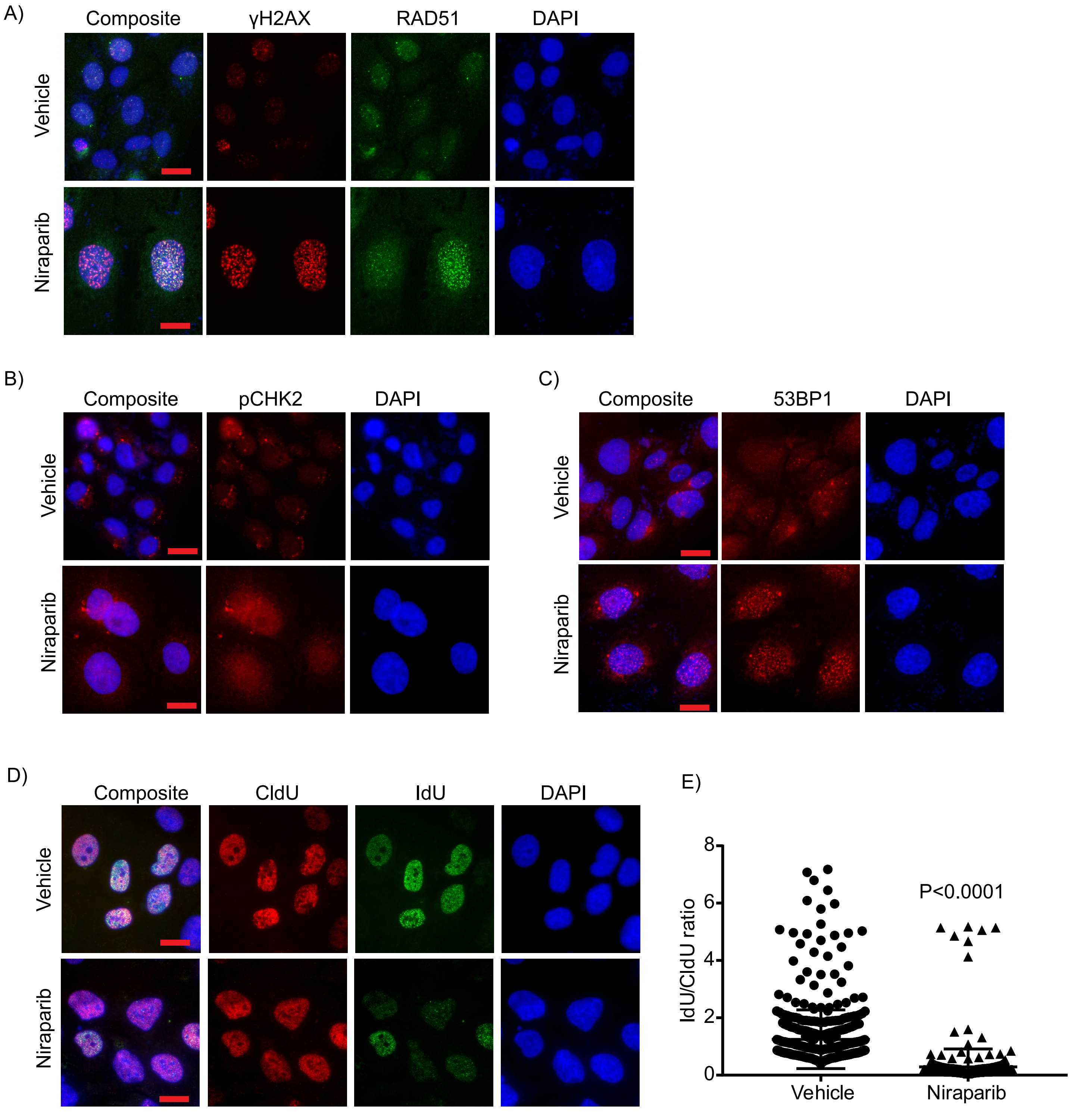

A)
Composite
γH2AX
RAD51
DAPI
Vehicle
Niraparib
B)
Composite
pCHK2
DAPI
Vehicle
Niraparib
C)
Composite
53BP1
DAPI
Vehicle
Niraparib
D)
Composite
CldU
IdU
DAPI
Vehicle
Niraparib
E)
IdU/CldU ratio
8
6
4
2
0
P<0.0001
Vehicle
Niraparib

Figure S5

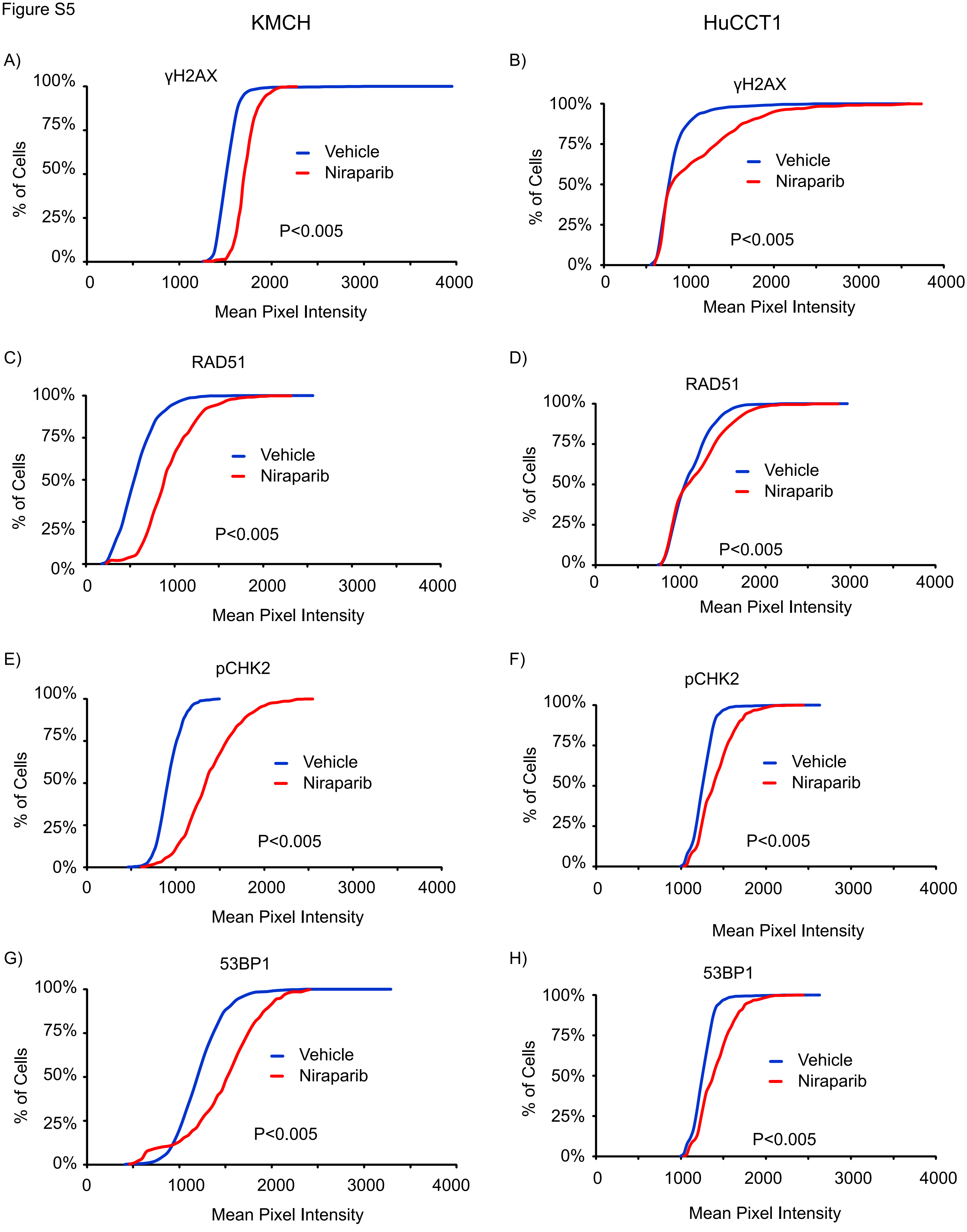
KMCH
HuCCT1
A)
γH2AX
100%
75%
50%
25%
0%
% of Cells
Vehicle
Niraparib
P<0.005
0
1000
2000
3000
4000
Mean Pixel Intensity
B)
γH2AX
Vehicle
Niraparib
P<0.005
Mean Pixel Intensity
C)
RAD51
Vehicle
Niraparib
P<0.005
Mean Pixel Intensity
D)
RAD51
Vehicle
Niraparib
P<0.005
Mean Pixel Intensity
E)
pCHK2
Vehicle
Niraparib
P<0.005
Mean Pixel Intensity
F)
pCHK2
Vehicle
Niraparib
P<0.005
Mean Pixel Intensity
G)
53BP1
Vehicle
Niraparib
P<0.005
Mean Pixel Intensity
H)
53BP1
Vehicle
Niraparib
P<0.005
Mean Pixel Intensity

Blots for Figure 2E, KMCH-1

Figure S6

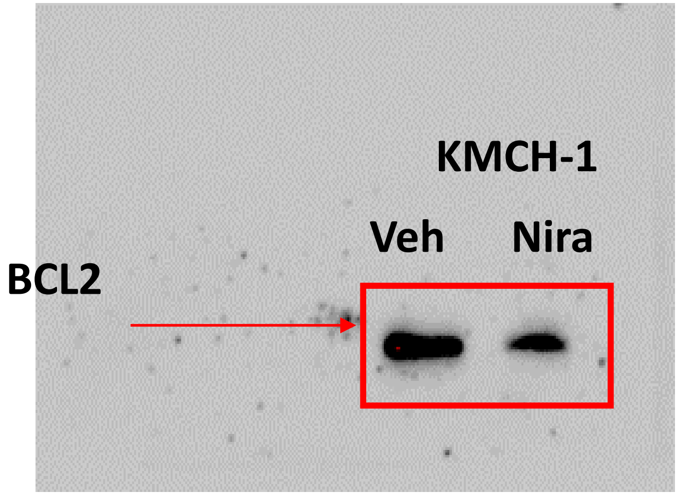

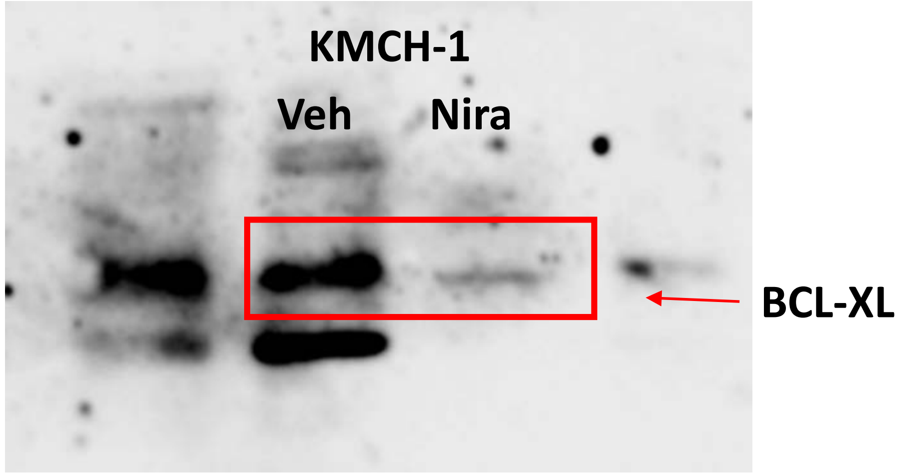

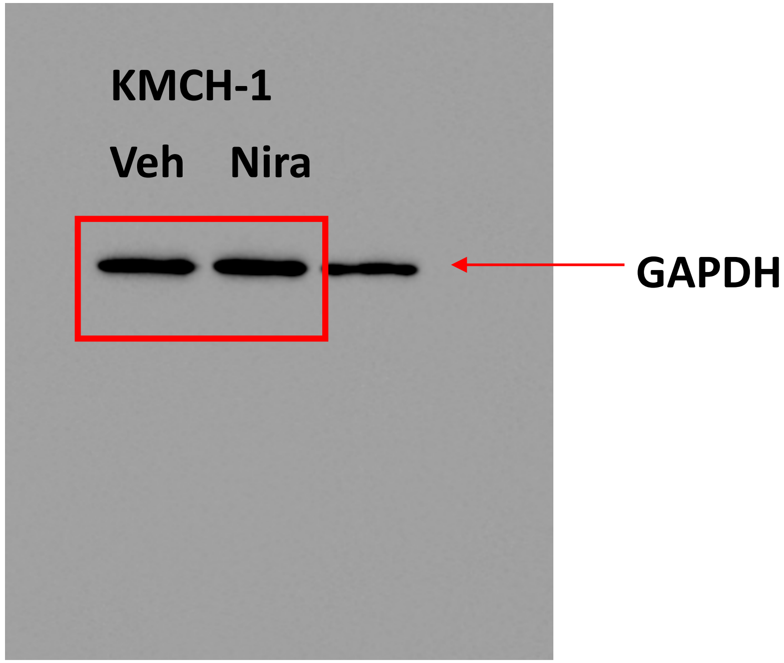

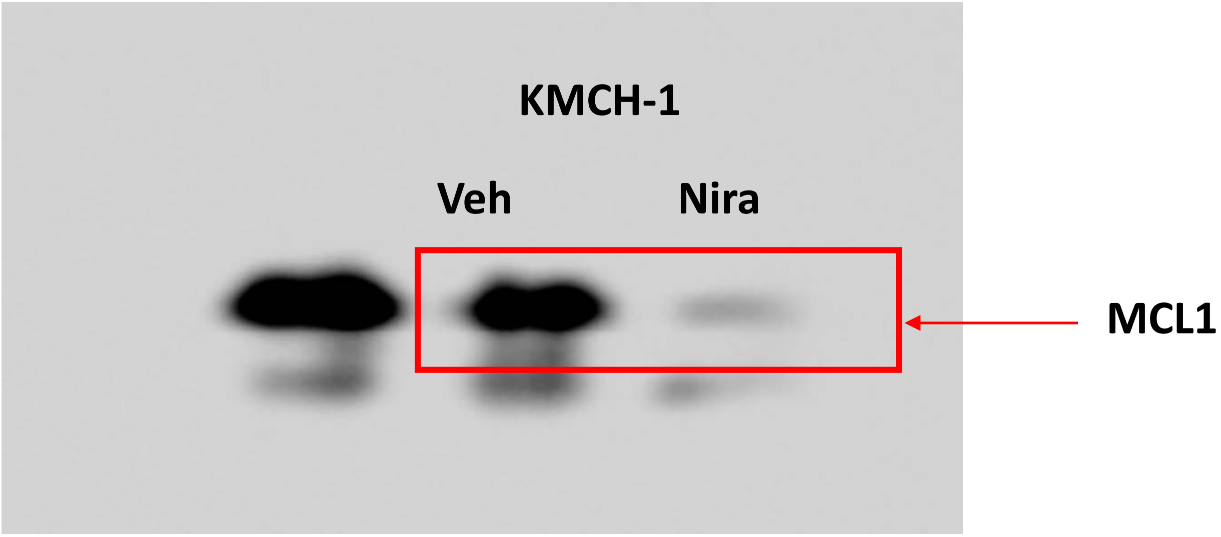

Figure S6

Blots for Figure 2F, HuCCT1

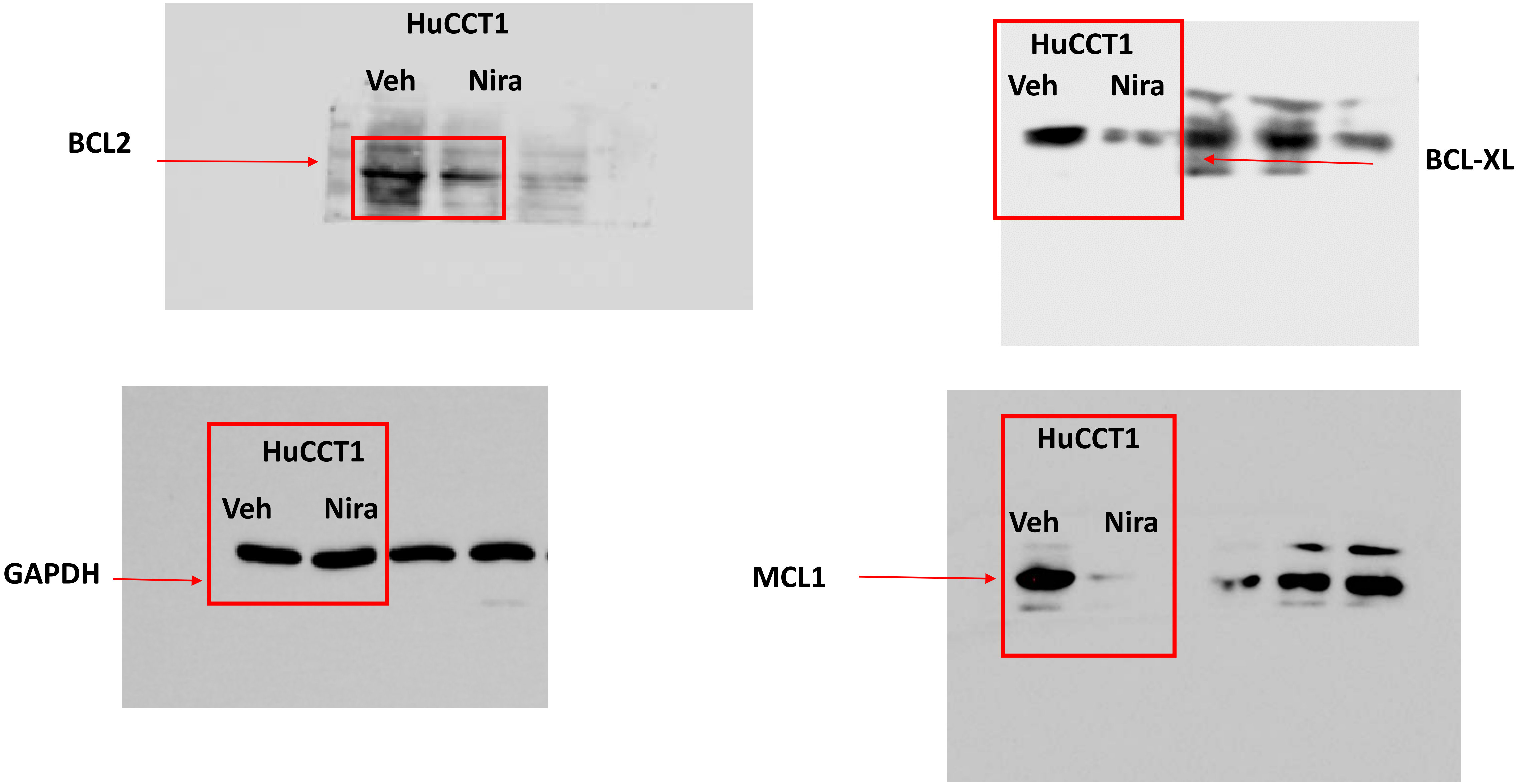

Figure S6

Blots for Figure 2G, CHNG6

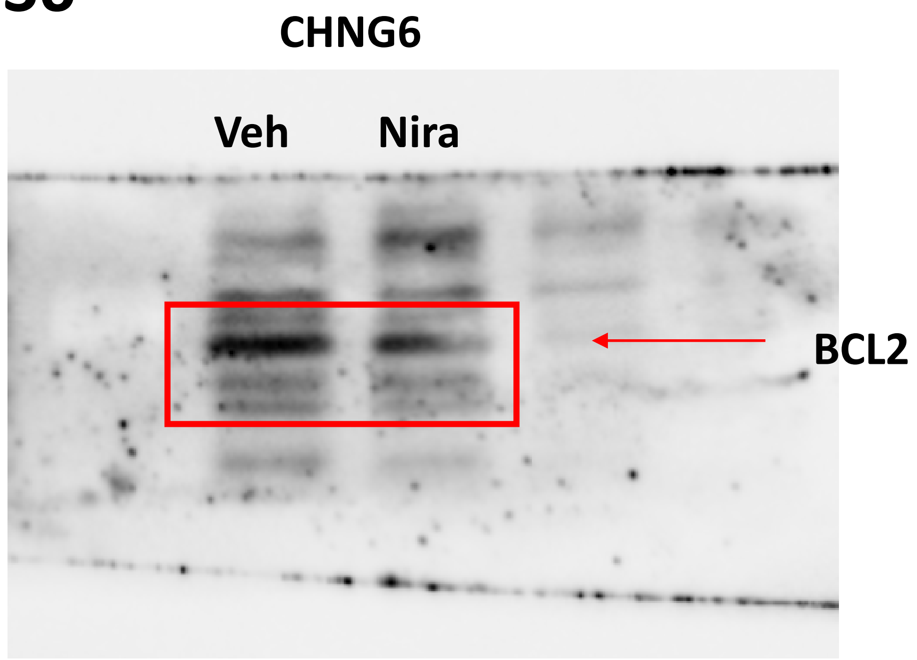

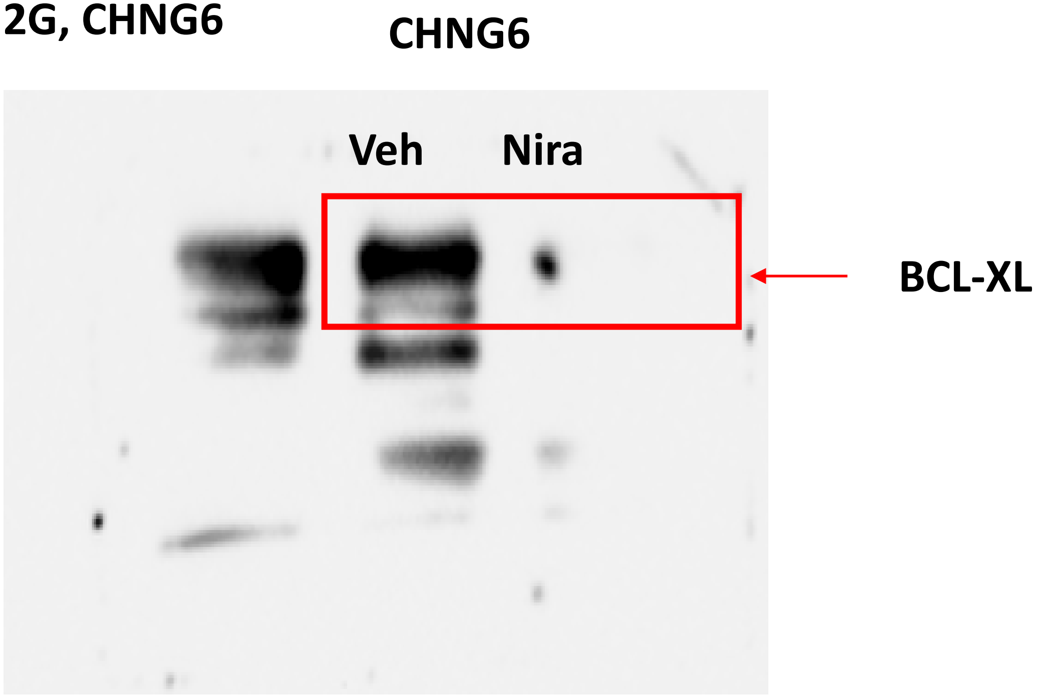

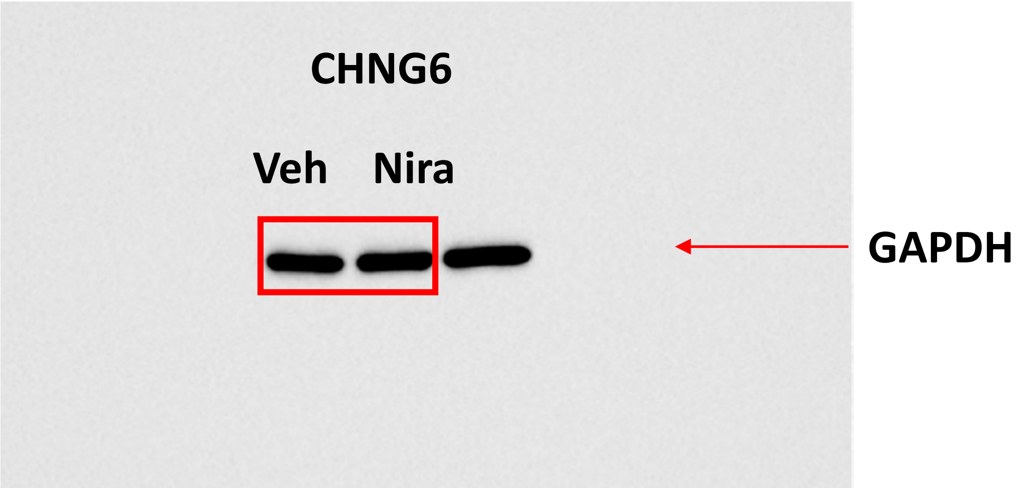

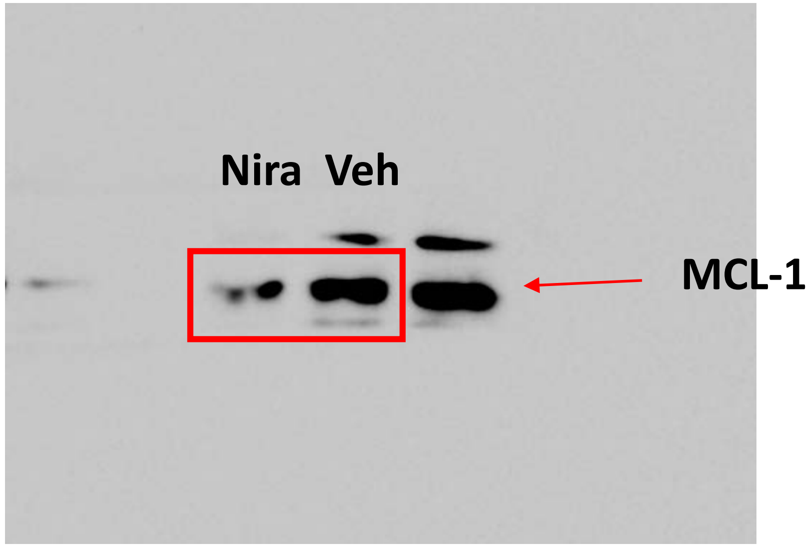

Figure S6

Blots for Figure 2H, CHNG31

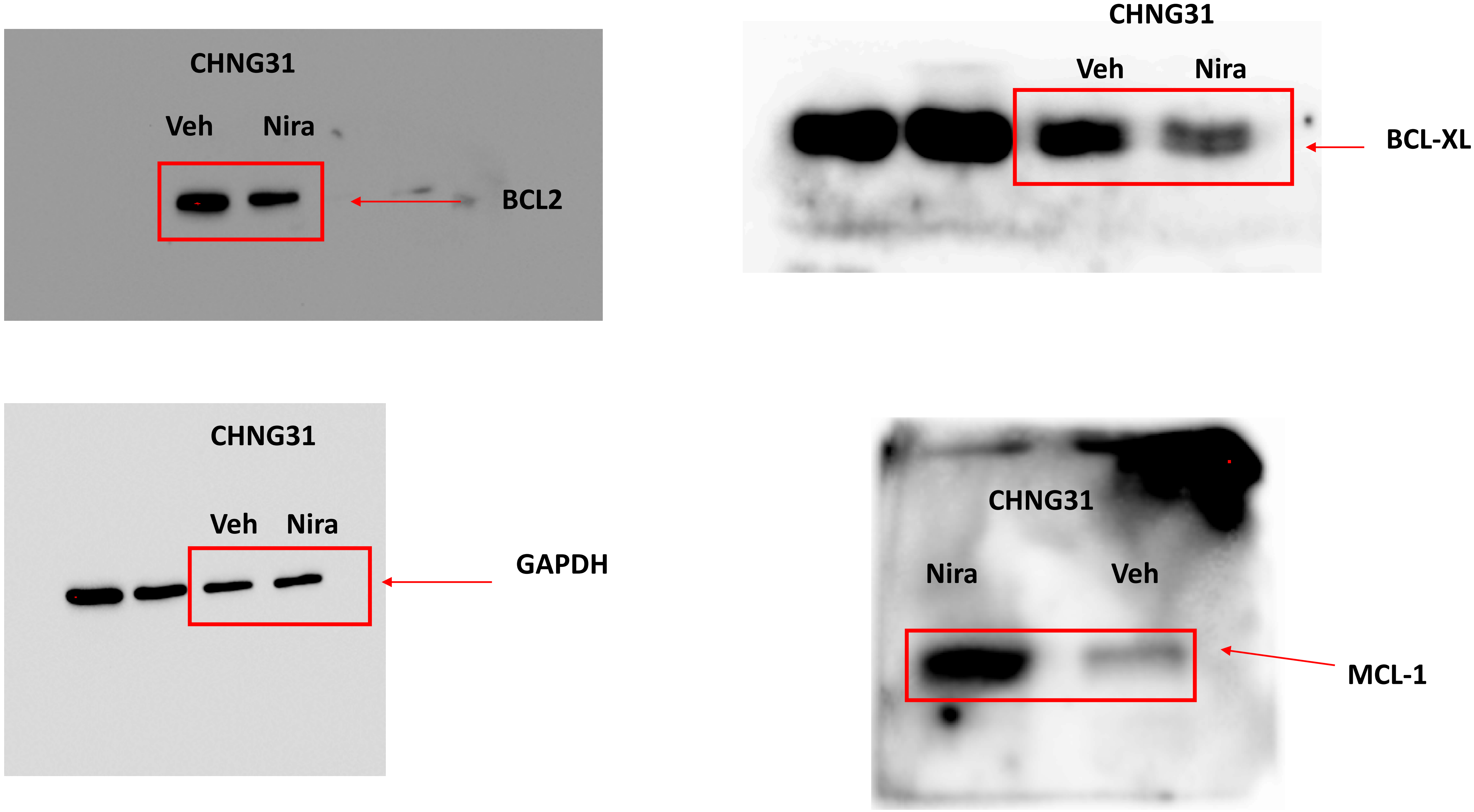

Figure S6

Blots for Figure 3C, KMCH-1

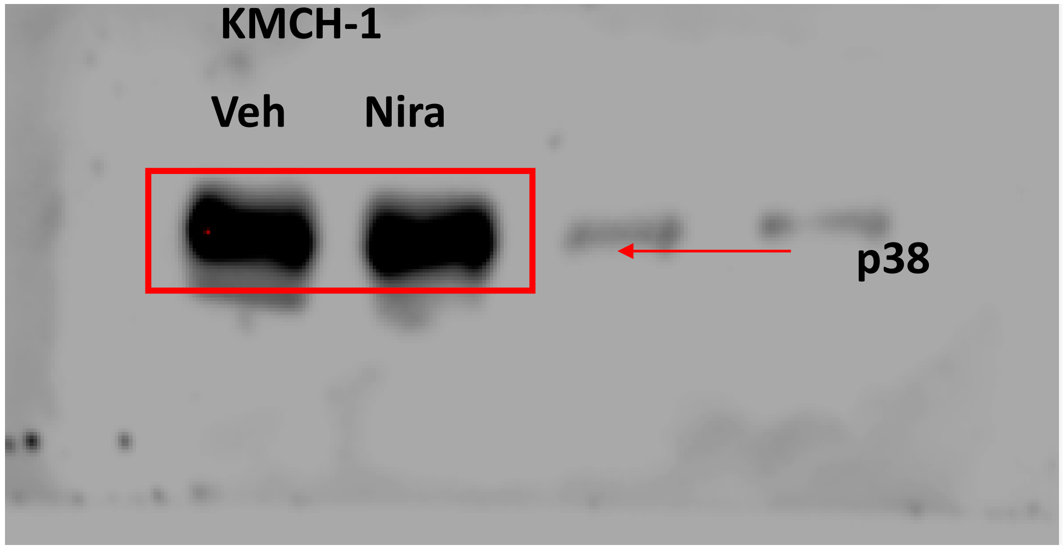

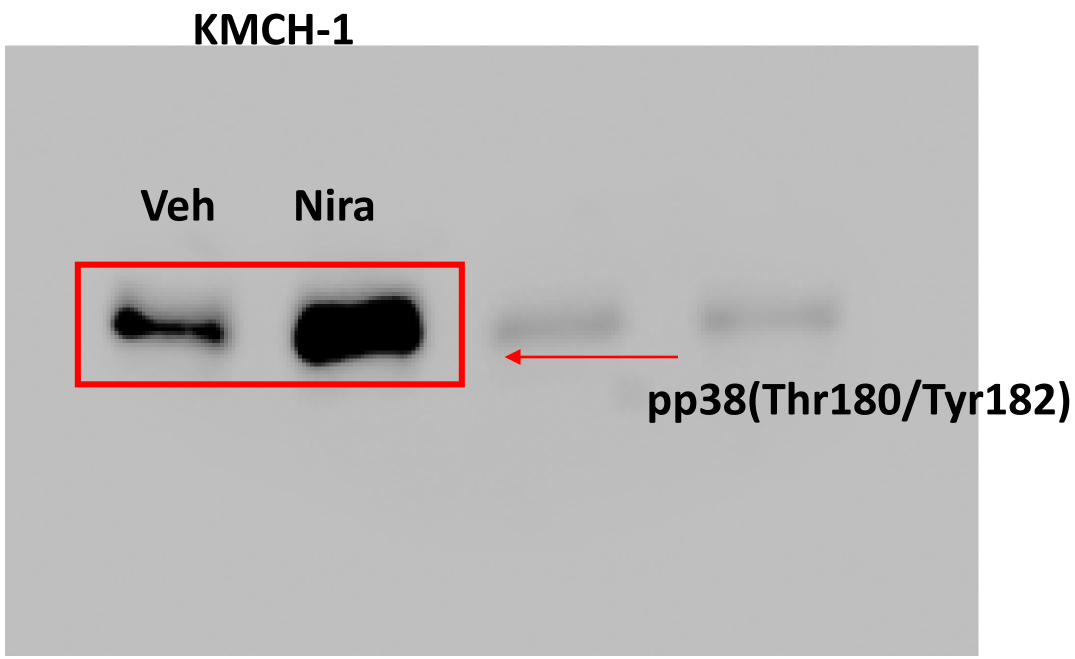

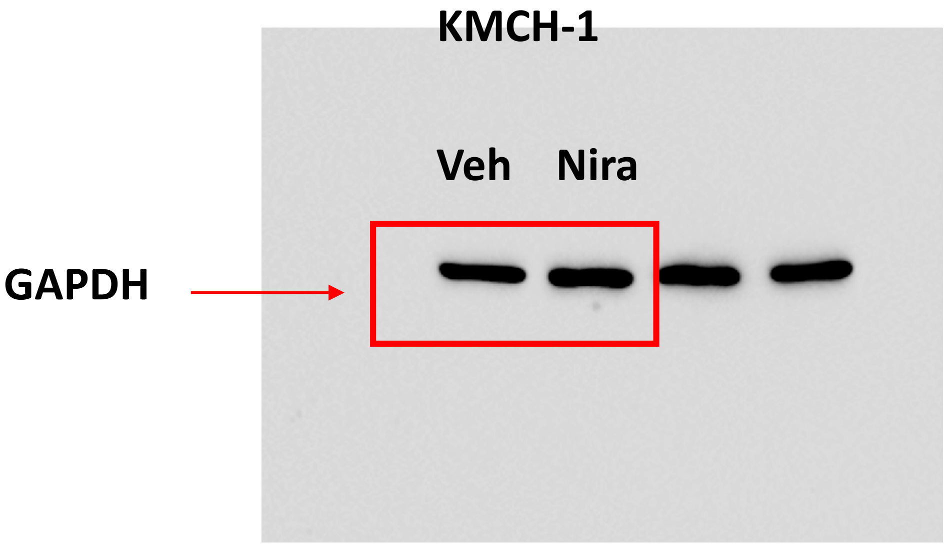

Figure S6

Blots for Figure 3D, HuCCT1

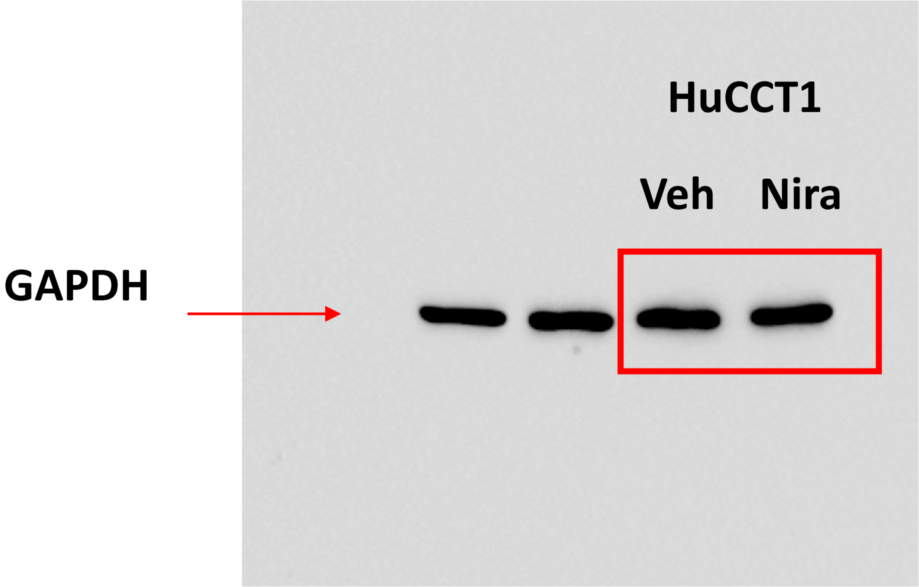

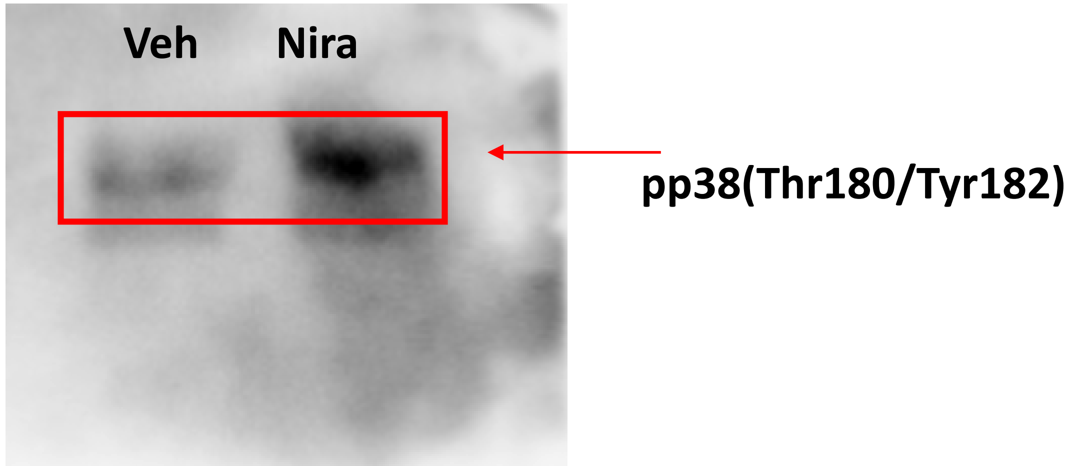

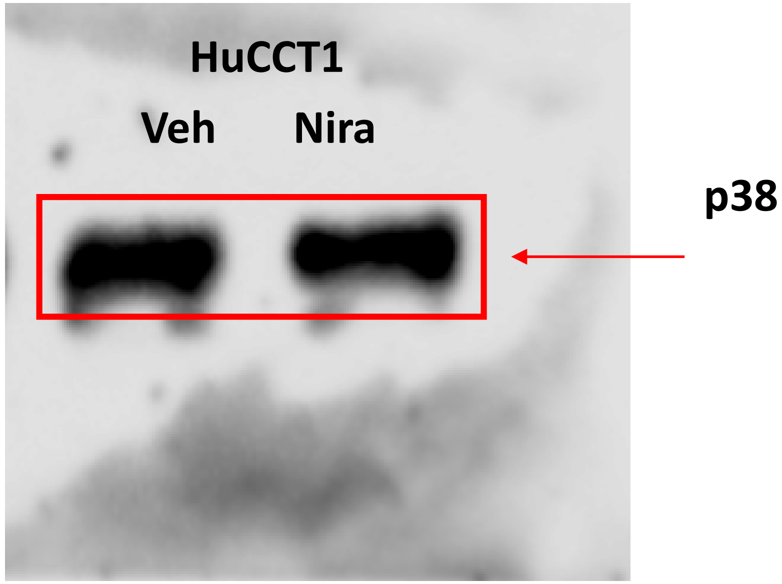

Figure S6

Blots for Figure 3E, CHNG6

GAPDH

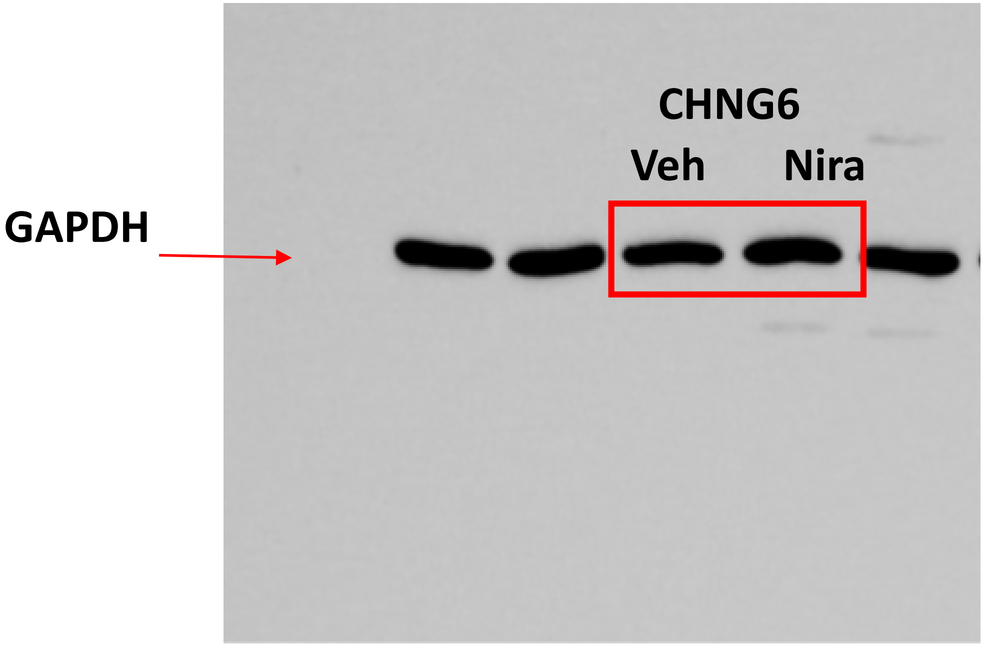

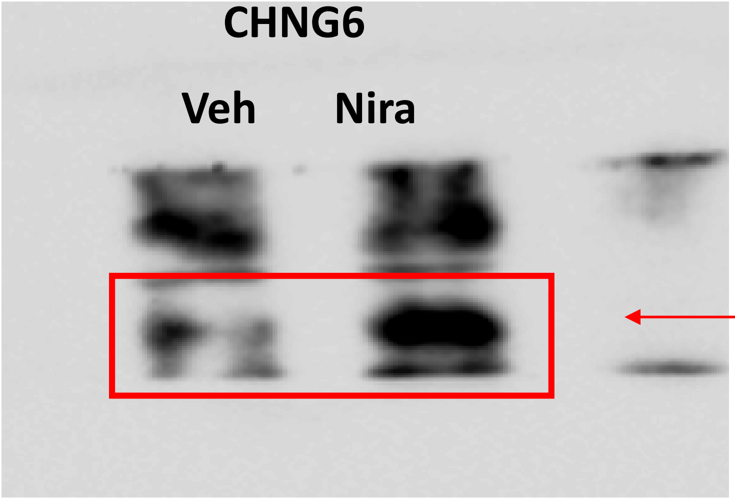

pp38 (Thr180/Tyr182)

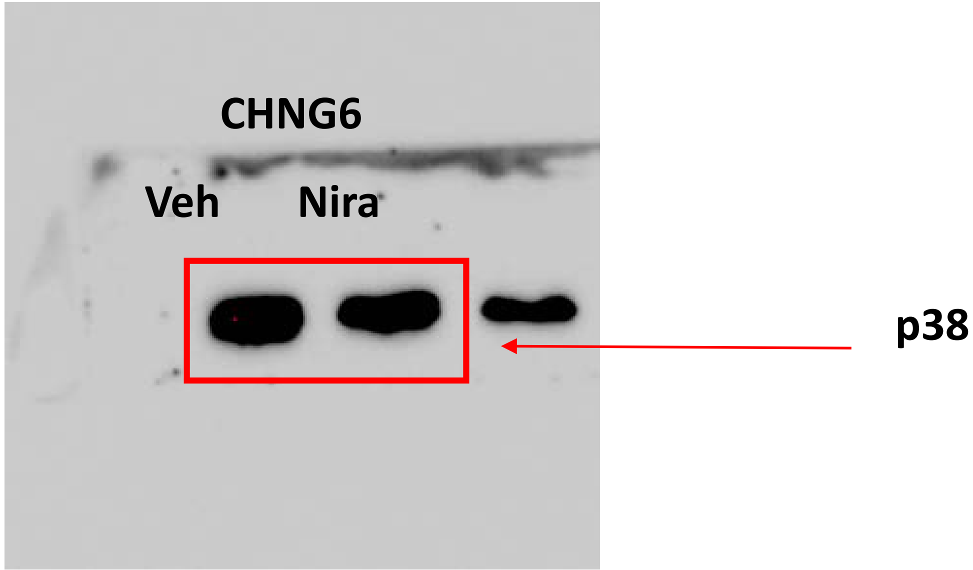

p38

Figure S6

Blots for Figure 3F, CHNG31

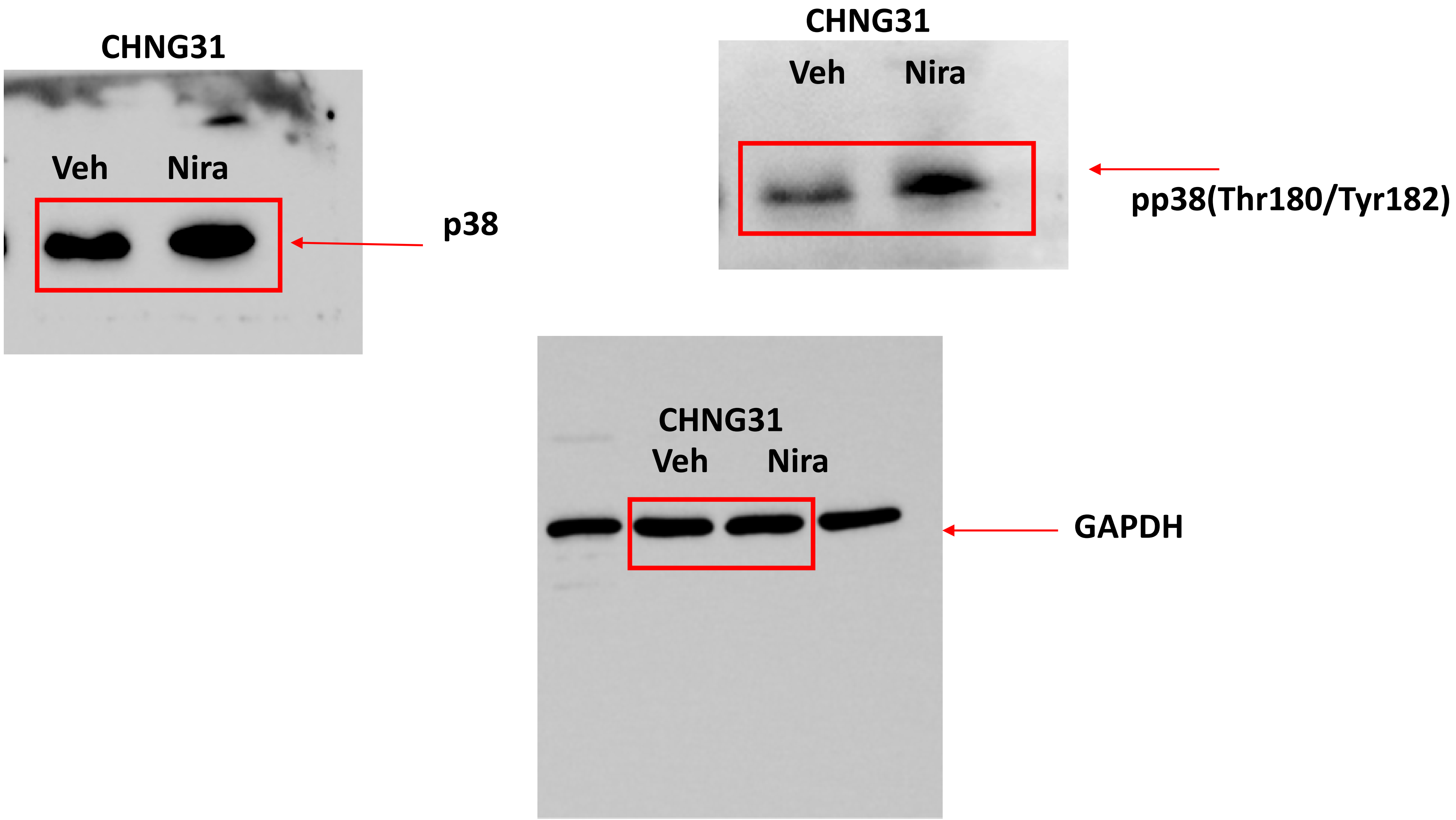

Figure S6

Blots for Figure 5B

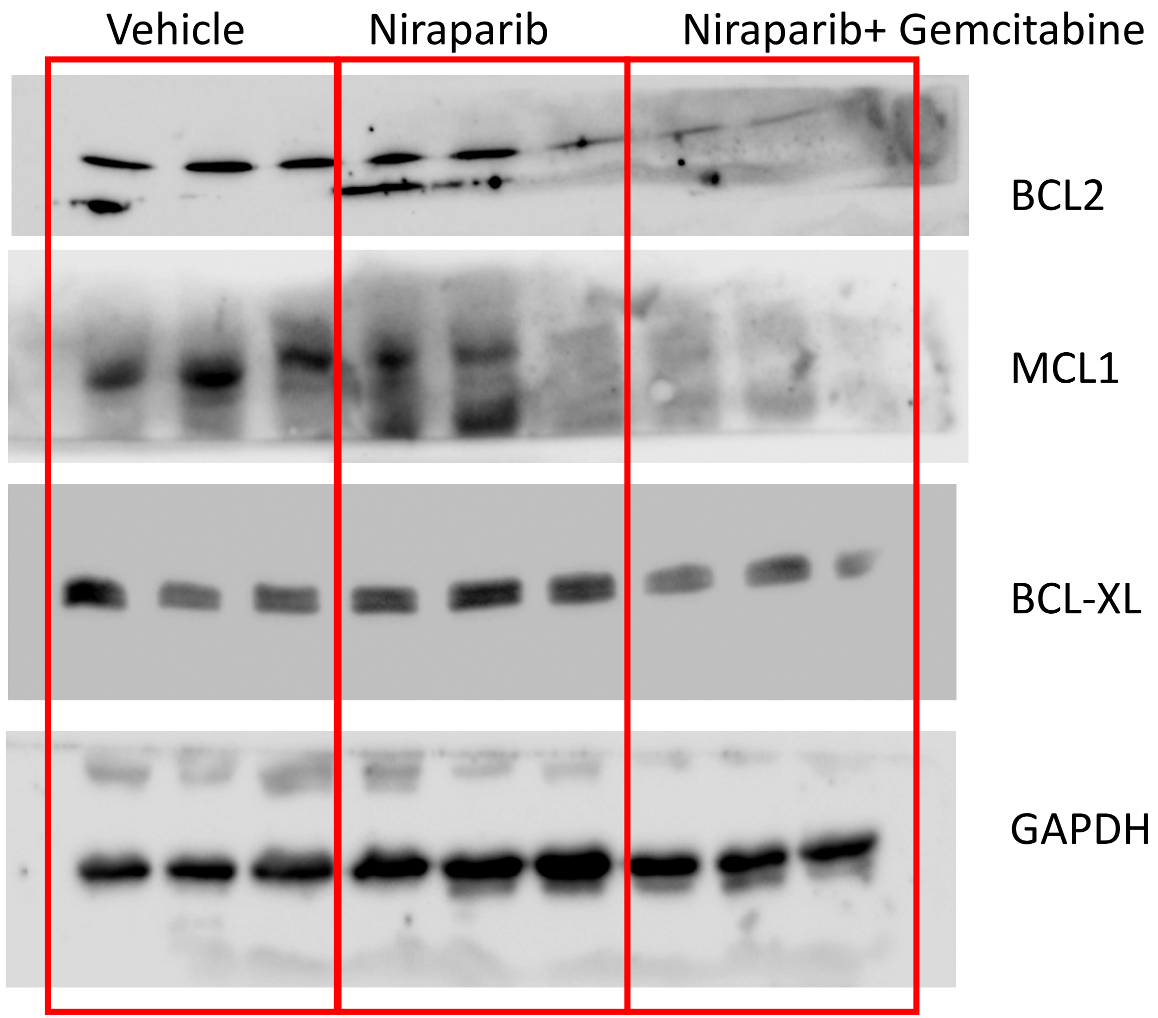

Supplement: Supplementary file 1 [file cancers-13-04405-s001.zip › cancers-1257541-supplementary.pdf]
